# Supplementary material for: Blood coagulation parameter abnormalities in hospitalized patients with confirmed COVID-19 in Ethiopia
Source: PLoS One. 2021 Jun 21;16(6):e0252939. doi: 10.1371/journal.pone.0252939 (PMC8216564; doi:10.1371/journal.pone.0252939)
Supplement: Attachment — Submitted filename: PONE-D-20-35898_reviewer SL.pdf [file pone.0252939.s001.pdf]

PLOS ONE

Blood coagulation parameter abnormalities among patients with confirmed COVID-19 in Ethiopia

--Manuscript Draft--

**Manuscript Number:**	PONE-D-20-35898
**Article Type:**	Research Article
**Full Title:**	Blood coagulation parameter abnormalities among patients with confirmed COVID-19 in Ethiopia
**Short Title:**	Coagulation profile of COVID-19 patients
**Corresponding Author:**	Shambel Araya, MSc<br>Addis Ababa University<br>Addis Ababa, ETHIOPIA
**Keywords:**	COVID-19; Prothrombin time; Activated partial thromboplastin time; international normalized ratio; Platelet; Addis Ababa; Ethiopia
**Abstract:**	Background<br><br>Infection with corona virus disease 2019 (COVID-19) could be complicated with coagulopathy and high risk of thromboembolic events. Currently abnormal coagulation profiles in COVID-19 patients are taken as an important prognostic factor of severity. The aim of the study was analyzing coagulation profile of admitted patients, with COVID-19 from Addis Ababa, Ethiopia.<br><br>Methods<br><br>This cross-sectional study was conducted among 455 Covid-19 patients admitted at Millennium COVID-19 treatment center Addis Ababa, Ethiopia from June 2020 to October 2020. Prothrombin Time (PT), Activated Partial Thromboplastin Time (APTT) and International normalized ratio(INR) were estimated by auto analyzer. In all tests, p<0.05 was defined as statistically significant.<br><br>Result<br><br>A prolonged ~~has been reported in up to~~ 46.8% of study subjects with COVID-19 ~~clinical conditions.~~ Prolonged prothrombin time and high INR ~~was~~ seen among 53.3% severe and 51% critical patients with COVID-19 manifestation. ~~An increase in coagulation parameters is the most significant change in coagulation parameters in severe COVID-19 patients, and progressively increasing values can be used as a prognostic parameter indicating a worse outcome in older peoples~~ (93 patients aged >55 years had a coagulopathy). Thrombocytopenia was detected in ~~around~~ 101/455 (22.1%) of individuals. 50.5% individual's aged older than 55 had thrombocytopenia.<br><br>Conclusion<br><br>We found an abnormal pattern of coagulation parameters and association of advanced age and co-morbidities with a high rate of mortality in severe COVID-19 patients, which should be taken into consideration in their hospital management.
**Order of Authors:**	Shambel Araya, MSc
	Mintesnot Aragaw Mamo
	Yakob Gebregziabher Tsegay
	Aschalew Aytenew
	Abebe Hordofa
	AbebeEdao Negeso
	Zemenu Tamir

Powered by Editorial Manager® and ProduXion Manager® from Aries Systems Corporation

	Tirhas Niguse
	Mahlet Cheru
	Asegdew Atlaw
**Additional Information:**	
**Question**	**Response**
**Financial Disclosure**<br><br>Enter a financial disclosure statement that describes the sources of funding for the work included in this submission. Review the submission guidelines for detailed requirements. View published research articles from *PLOS ONE* for specific examples.<br><br>This statement is required for submission and **will appear in the published article** if the submission is accepted. Please make sure it is accurate.<br><br>**Unfunded studies**<br>Enter: *The author(s) received no specific funding for this work.*<br><br>**Funded studies**<br>Enter a statement with the following details:<br>• Initials of the authors who received each award<br>• Grant numbers awarded to each author<br>• The full name of each funder<br>• URL of each funder website<br>• Did the sponsors or funders play any role in the study design, data collection and analysis, decision to publish, or preparation of the manuscript?<br>• **NO** - Include this sentence at the end of your statement: *The funders had no role in study design, data collection and analysis, decision to publish, or preparation of the manuscript.*<br>• **YES** - Specify the role(s) played.<br><br>* typeset	The author(s) received no specific funding for this work.
**Competing Interests**<br><br>Use the instructions below to enter a competing interest statement for this submission. On behalf of all authors, disclose any competing interests that could be perceived to bias this	The authors have declared that no competing interests exist

work—acknowledging all financial support and any other relevant financial or non-financial competing interests.<br><br>This statement **will appear in the published article** if the submission is accepted. Please make sure it is accurate. View published research articles from *PLOS ONE* for specific examples.<br><br>**NO authors have competing interests**<br><br>Enter: *The authors have declared that no competing interests exist.*<br><br>**Authors with competing interests**<br><br>Enter competing interest details beginning with this statement:<br><br>*I have read the journal's policy and the authors of this manuscript have the following competing interests: [insert competing interests here]*<br><br>* typeset	
**Ethics Statement**<br><br>Enter an ethics statement for this submission. This statement is required if the study involved:<br><br>• Human participants<br>• Human specimens or tissue<br>• Vertebrate animals or cephalopods<br>• Vertebrate embryos or tissues<br>• Field research<br><br>Write "N/A" if the submission does not require an ethics statement.<br><br>General guidance is provided below. Consult the submission guidelines for detailed instructions. **Make sure that all information entered here is included in the Methods section of the manuscript.**	Addis Ababa University College of Health Science Department of Medical Laboratory Science (DRERC) protocol number: DRERC/469/19/MLS

**Format for specific study types**<br><br>**Human Subject Research (involving human participants and/or tissue)**<br>• Give the name of the institutional review board or ethics committee that approved the study<br>• Include the approval number and/or a statement indicating approval of this research<br>• Indicate the form of consent obtained (written/oral) or the reason that consent was not obtained (e.g. the data were analyzed anonymously)<br><br>**Animal Research (involving vertebrate animals, embryos or tissues)**<br>• Provide the name of the Institutional Animal Care and Use Committee (IACUC) or other relevant ethics board that reviewed the study protocol, and indicate whether they approved this research or granted a formal waiver of ethical approval<br>• Include an approval number if one was obtained<br>• If the study involved *non-human primates*, add *additional details* about animal welfare and steps taken to ameliorate suffering<br>• If anesthesia, euthanasia, or any kind of animal sacrifice is part of the study, include briefly which substances and/or methods were applied<br><br>**Field Research**<br><br>Include the following details if this study involves the collection of plant, animal, or other materials from a natural setting:<br>• Field permit number<br>• Name of the institution or relevant body that granted permission	
**Data Availability**<br><br>Authors are required to make all data underlying the findings described fully available, without restriction, and from the time of publication. PLOS allows rare exceptions to address legal and ethical concerns. See the PLOS Data Policy and FAQ for detailed information.	Yes - all data are fully available without restriction

A Data Availability Statement describing where the data can be found is required at submission. Your answers to this question constitute the Data Availability Statement and **will be published in the article**, if accepted.<br><br>**Important:** Stating 'data available on request from the author' is not sufficient. If your data are only available upon request, select 'No' for the first question and explain your exceptional situation in the text box.<br><br>Do the authors confirm that all data underlying the findings described in their manuscript are fully available without restriction?	
**Describe where the data may be found in full sentences. If you are copying our sample text, replace any instances of XXX with the appropriate details.**<br><br>• If the data are **held or will be held in a public repository**, include URLs, accession numbers or DOIs. If this information will only be available after acceptance, indicate this by ticking the box below. For example: *All XXX files are available from the XXX database (accession number(s) XXX, XXX.).*<br>• If the data are all contained **within the manuscript and/or Supporting Information files**, enter the following: *All relevant data are within the manuscript and its Supporting Information files.*<br>• If neither of these applies but you are able to provide **details of access elsewhere**, with or without limitations, please do so. For example:<br><br>*Data cannot be shared publicly because of [XXX]. Data are available from the XXX Institutional Data Access / Ethics Committee (contact via XXX) for researchers who meet the criteria for access to confidential data.*<br><br>*The data underlying the results presented in the study are available from (include the name of the third party*	The data underlying the results presented in the study are available from the corresponding author in a reasonable request

and contact information or URL).<br>• This text is appropriate if the data are owned by a third party and authors do not have permission to share the data.<br><br>* typeset	
Additional data availability information:	

manuscript Click here to access/download;Manuscript;COP manuscript Plos One.docx

Blood coagulation parameter abnormalities ~~among~~ patients with confirmed COVID-19 in Ethiopia

Shambel Araya[1&2], Mintesnot Aragaw Mamo[2&3], Yakob Gebregziabher Tsegay[2&4], Aschalew Aytenew[2&3], Abebe Hordofa[2], AbebeEdao Negeso[1], MogesWordofa[1], Zemenu Tamir[1], Tirhas Niguse[1], Mahlet Cheru[1], Asegdew Atlaw[2]

1. Department of Medical Laboratory Sciences, College of Health Sciences, Addis Ababa University, Addis Ababa, Ethiopia
2. Millennium COVID-19 treatment center, Addis Ababa, Ethiopia
3. St. Paul hospital millennium medical college, Addis Ababa, Ethiopia
4. College of Health Science, Defense University Addis Ababa, Ethiopia

Adress:

1. Shambel Araya(corresponding author); shambelaraya8@gmail.com

2: Mintesnot Aragaw Mamo: mintsh2015@gmail.com

3: Yakob Gebreegziabher Tsegaye: yakobtsegay17@gmail.com

4: Aschalew Aytenew: aschu9033@gmail.com

5: Abebe Hordofa: abuhordofa@gmail.com

6: Abebe Edao Negeso: abenegesso@gmail.com

7: Moges Wordofa: heranmakmow@gmail.com

8: Zemenu Tamir: zemenut266@gmail.com

9: Tirhas Niguse; peace.for.all.060610@gmail.com

10. Mahlet Cheru: yuluyaya54@gmail.com

11.Asegdew Atlaw: asegdew21@gmail.com

Abstract

Background: Infection with corona virus disease 2019 (COVID-19) could be complicated with coagulopathy and high risk of thromboembolic events. Currently abnormal coagulation profiles in COVID-19 patients are taken as an important prognostic factor of severity. The aim of the study was analyzing coagulation profile of admitted patients, with COVID-19 from Addis Ababa, Ethiopia. **Methods**: This cross-sectional study was conducted among 455 Covid-19 patients admitted at Millennium COVID-19 treatment center Addis Ababa, Ethiopia from June 2020 to October 2020. Prothrombin Time (PT), Activated Partial Thromboplastin Time (APTT) and International normalized ratio(INR) were estimated by auto analyzer. In all tests, $p<0.05$ was defined as statistically significant. **Result**: A prolonged has been reported in up to 46.8% of study subjects with COVID-19 clinical conditions. Prolonged prothrombin time and high INR was seen among 53.3% severe and 51% critical patients with COVID-19 manifestation. An increase in coagulation parameters is the most significant change in coagulation parameters in severe COVID-19 patients, and progressively increasing values can be used as a prognostic parameter indicating a worse outcome in older peoples (93 patients aged >55 years had a coagulopathy). Thrombocytopenia was detected in around 101/455 (22.1%) of individuals. 50.5% individual's aged older than 55 had thrombocytopenia.

Conclusion: We found an abnormal pattern of coagulation parameters and association of advanced age and co-morbidities with a high rate of mortality in severe COVID-19 patients, which should be taken into consideration in their hospital management.

Key words:COVID-19, Prothrombin time, Activated partial thromboplastin time, international normalized ratio, Platelet, Addis Ababa, Ethiopia.

Introduction

Coronavirus disease 2019 (COVID-19) is caused by a novel beta coronaviruscalled as severe acute respiratory syndrome coronavirus 2 (SARS-CoV-2)(1). COVID-19 has become a pandemic that has heavily affected the global population. As of November 8,2020,there have been 49,578,590 confirmed cases of COVID-19 and 1,245,717 deaths, reported to WHO. Similarly, there have been 99,204 confirmed cases of COVID-19 with 1,518 deaths in Ethiopia(2).

The severity of COVID-19 varies considerably from asymptomatic to life threatening, lung injury being the main clinical manifestation. Most of the patients have a favorable prognosis, but some rapidly progress to severe and critical cases with respiratory distress syndrome, coagulation dysfunction and multiple organ failures(3, 4).Althoughthe pathophysiology and the underlining mechanisms of clinical manifestations remain unclear,thrombo-inflammation and cytokine storm are clearly established components in Severe Acute Respiratory Distress Syndrome (SARS) of COVID-19(5-8).

Coagulopathy and abnormal coagulation factorswere indicated among the most significant markers of poor prognosis in COVID-19 (9-11). A retrospective cohort study conducted in Spain Madrid showed COVID 19 non-survivors hadsignificantly lower prothrombin activity, abnormal coagulation parameters like prolonged PT, APTT, higher D- dimer, higher fibrinogen levels compared to survivors indicating coagulation parameters could be an efficient measure for improving the clinical management and predicting the prognosis of patients with SARS COV-2(7).Similarly, Long et al has reported that occurrence of coagulation dysfunction is more likely in severe and critically ill patients. The study also showed that D-dimer and prothrombintime could be considered as a main indicators in predicting the mortality of COVID-19(3). Different

studies also support that patients infected by COVID-19 are at high risk of developing disseminated intravascular coagulation (12, 13).

It also indicated that comparison of reports from various populations related to the clinical course, outcome of COVID-19 and blood coagulation profile in these patients are necessary to help the management and treatment of the disease(12, 14). Moreover, this routine coagulation parameter tests could be used as potential indicators for COVID-19 in individuals having typical clinical manifestations that would be inputs for prompt patient management especially in resource limited settings where the high-tech gold standard RT-PCR is not widely available, like Ethiopia. However, data on coagulation profiles among Ethiopian COVID-19 patients is scarce. Thus, the aim of this study was to find out the coagulation profile of COVID-19 patients admitted at Millennium COVID-19 treatment center, Addis Ababa, Ethiopia.

Methods

Study population

In this study, we have included 455 consecutive patients with confirmed SARS-CoV-2 infection and admitted to Millennium COVID-19 treatment center Addis Ababa, Ethiopia from July 1-October 23, 2020. The treatment center is the first referral center of COVID-19 patients in Ethiopia, since May 02, 2020. Blood samples were collected by nurse professionals in the treatment center and transported to Millennium COVID-19 treatment center. None of the study participants was taking anticoagulant drugs before blood drawing. Diagnosis of COVID-19 was made according to real time PCR.

Sample collection and coagulation profile analysis

2.6. Laboratory Analysis

Eight milliliters of venous blood were collected: five milliliters in EDTA for platelet count, three milliliters in 3.2% sodium citrated anti-coagulated tube for analysis of coagulation parameters. The samples for coagulation tests were collected at hospital admission. The prothrombin time (PT), activated partial prothrombin time (APTT), and international normalized ratio (INR) were analyzed using HUMACLOT DUE PLUS [illegible]agulation analyzer (Wiesbaden, Germany). Platelet count was performed using Beckman [illegible]lter DxH 600 automated hematology analyzer. The coagulation parameters were compared with the manufacturer cutoff normal range of PT = 11.7- 15 seconds, APTT = 23.8- 37.9 seconds, INR = 1.0- 1.2 and PLT= 159-386/µ.l. The coagulation parameters above the cutoff range were considered as a prolonged time and thrombocytopenia in the case of lower than cut value for platelet. All laboratory tests and interpretation were done following the manufacturers' recommendation and standard operating procedure.

Ethical Consideration. The study was approved by Addis Ababa University College of health science, department of medical laboratory ethics and research committee. Informed consent was taken from each of the study participants. Laboratory test results were communicated to the responsible clinicians working at the treatment center. All the information obtained from the study participants were kept confidential.

Statistical Analysis

SPSS statistical software package version 25.0 (SPSS Inc., Chicago, IL, USA) was used for statistical analysis. Chi-square test was used to determine association among categorical variables. The quantitative data were expressed as Mean±SD. P-value < 0.05 was considered as statistically significant.

Results

Socio-demographic and Clinical characteristics of Study participants

In this study, 455 patients diagnosed with COVID-19 were included. Among they study participants, 289 (63.5%) were males. The study participants were between the age of 4-90 with a mean of 49.9 ±18.3 years. From the total 455 case, there were 297 Mild case, 90 severe cases, and 68 critical cases. These cases were divided into three groups based on the disease severity and the rate of critical cases was 15%(Table 1).

Table 1: Socio-demographic characteristics of study participants Addis Ababa, Ethiopia, 2020

Variables		Frequency	Percent
Gender	Male	289	63.5%
	Female	166	36.5%
Age group	0-18 years	15	3.2%
	18-35 years	101	22.1%
	36-55 years	158	34.7%
	>55 years	181	39.7%
Disease severity	Moderate	297	65.2%
	Severe	90	19.8%
	Critical	68	15%

The median time from the disease onset to admission was 4 days (2-8 days). Severe and critical groups showed statistically significant differences in sex ratio and age distribution. In severeand critical groups, majority were males and elderly (Table 1

Table 1: Socio-demographic characteristics and disease severity of COVID-19 patients admitted to Millennium COVID-19 treatment center Addis Ababa, Ethiopia

Variables		Disease Severity			P-value
		Moderate n(%)	Severe	Critical n=68	
Age(year)	0-18 n= 15	10	4	1	0.283
	18-35 n=101	65	22	14	
	36-55 n=158	107	31	20	
	>55 n=181	115	33	33	
Sex N=455	Male n=289	187	56	46	0.045
	Female n=166	110	34	22	

Magnitude of coagulation abnormalities

In this study, 209 COVID-19 patients (46%) showed prolonged PT and higher INR values. Among those patients with prolonged PT, 51.3% are above 55 years. Prolonged PT value was

found among males (49.8%) than females (41%)and prolonged PT has a significant association with gender(P-value 0.045). Similarly, 51.4% and 53.3% ICU (critical) and severe patients had a prolonged PT value. Notably, prolonged APTT values were found among 196/455 individuals and from these 47%, 45% and 41% were the distribution among ICU (critical), Severe and moderate patients respectively.95/166 female patients had prolonged APTT and 51.3% patients aged older than 55 years had a prolonged APTT. Thrombocytopenia and abnormal coagulation parameters (PT, APTT and INR) could be considered as important indicators of COVID-19 disease severity.

Thrombocytopenia was detected in around 101/455 (22.1%) of individuals. 50/99 (50.5%) individual's aged older than 55 had thrombocytopenia. Thrombocytopenia was higher among male (23.8%) ICU patients than female(18%) ICU patients.

Table 2: Coagulation parameters in patients with severe COVID-19 according to different variables

Coagulation Parameters		***Variables***								
		Age				***Sex***		***Disease Severity***		
		0-18 n=15	***19-35 n=101***	***36-55 n=158***	***>55 n=181***	***Male n=289***	***Female n=166***	***Moderate n=297***	***Severe n=90***	***Critical n=68***
PT	High	9	50	61	93	144	69	130	48	35
	Normal	6	45	89	80	131	89	149	40	31
	Low	0	6	8	8	14	8	18	2	2

APTT	High	6	46	68	76	101	95	115	41	42
	Normal	6	38	70	79	136	57	137	36	21
	Low	3	17	20	26	52	14	45	13	5
PLT	High	4	11	24	26	43	22	39	8	8
	Normal	9	70	107	105	177	114	130	44	31
	Low	2	20	27	50	69	30	128	38	29
INR	High	8	52	60	92	140	68	96	49	33
	Normal	6	39	88	77	105	65	106	38	32
	Low	1	10	10	12	44	33	95	3	3

PLT=platelet; PT= prothrombin time; APTT=activated partial thromboplastin time; INR=international normalized ratio.

Discussion

COVID-19, which is caused by SARS-CoV-2, has spread across the globe. Although most patients recover within 1 to 3 weeks, COVID-19 has already caused >1.2 million deaths worldwide and more than 1400 in Ethiopia as of October 30,2020 according to WHO report(15). Dysregulation of coagulation produces a coagulopathy associated with hyper coagulability as evidenced by venous and arterial thrombosis and multi-organ dysfunction. Up to 20% of affected patients require hospitalization, and the mortality rate in such patients is high(16, 17). Coagulopathy is one of the most significant prognostic factors in patients with COVID-19 and is

associated with increased mortality and admission to critical care(14, 18). Most commonly observed coagulopathy in patients hospitalized with COVID-19 (COVID-19-associated coagulopathy) is characterized by increased coagulation parameters like PT, APTT and INR levels(19, 20).

Patients with a serious infection are more likely to have COVID-19 associated coagulopathy than patients with a mild infection (21,22). In our study prolonged PT, APTT an INR was found among Severe and critical patients than moderate clinical manifestations and many studies also report that thrombotic complications are common among patients admitted to the intensive care unit (ICU) for COVID-19 (reported in 9.5%-47%(21-23).

As for all coagulopathies, treatment of the underlying condition is paramount. Experience to date suggests that COVID-19 infection infrequently leads to bleeding despite abnormal coagulation parameters (23,24). Supportive care including blood product transfusion should be individualized(24, 25). Blood component therapy should not be instituted on the basis of laboratory results alone, but reserved for those who are bleeding, require an invasive procedure, or who are otherwise at high risk for bleeding complications(25).

Considerable evidence indicates that COVID-19 is associated with a hyper-coagulable state. Thus, despite anticoagulant thrombo-prophylaxis, different studies have reported that rates of venous thromboembolism (VTE) and arterial thrombosis ranging from 15% to 30% in critically ill patients with COVID-19 and ~7% in those admitted to medical wards(26-28).Clotting of access catheters, dialysis membranes, and extracorporeal circuits has also been reported. Furthermore, in patients dying from COVID-19, autopsy studies reveal unsuspected deep vein thrombosis and multiple thrombi in the vessels of the lungs, kidneys, and other organs(9, 27). These findings have prompted some clinicians to use treatment doses of heparin or low-

molecular-weight heparin instead of prophylactic doses in critically ill COVID-19 patients(12, 25, 29).

In this current study, thrombocytopenia was observed among males(23.8%) than males(19.8%) and older peoples(27.6%). Severe(42.68%) and critical (42%) patients had also shown thrombocytopenia and this was in line with studies conducted in different countries (19, 21, 30, 31). Thrombocytopenia, defined as platelet count less than 100×10^9 cells per L were independently associated with severity. Studies across suggested that routine coagulation tests can be considered as a significant marker to help clinicians assess prognosis and severity of patients with COVID-19.In critically ill patients, thrombocytopenia correlates with multi-organ failure and death, and a decline in platelet count, even in the absence of overt thrombocytopenia, portends a worse outcome(9, 12, 13)In patients who are not bleeding, there is no evidence that correction of laboratory parameters with blood products improves outcomes. Replacement might worsen disseminated thrombosis and further deplete scarce blood products(26, 32).

As many studies reported that the coagulopathy associated with COVID-19 is characterized by thrombocytopenia, prolongation of the prothrombin time, high levels of D-dimer, and elevated levels of fibrinogen, factor VIII, and von Willebrand factor. The levels of coagulation correlate with disease severity and predict the risk of thrombosis, the need for ventilator support, and mortality. Although the features of COVID-19-associated coagulopathy have been considered unique with avery decreased platelet count(9, 33-35). However, in patients with critical COVID-19 infection and a cytokine storm, an extreme hyper-coagulable state was reported. Even though the reason for this life-threatening condition is not known but this might be due to an uncontrolled hyper-inflammatory response without previous immunity(36, 37).

Conclusion: We recommend monitoring platelet count, PT, APTT and INR. Worsening of these parameters indicates progressive severity of COVID-19 infection and predicts that more aggressive critical care will be needed; experimental therapies for COVID-19 infection might be considered in this setting. Improvement of coagulationparameters along with improving clinical condition provides confidence that stepping down of aggressive treatment may be appropriate

EthicalClearance: Ethical clearance was obtained from Addis Ababa University, College of Health Science, Department of Medical Laboratory Science ethical review committee and it was in accordance with the principles of the Helsinki II declaration.

Consent for publication: Not applicable

Availability of data and material

The data sets used or analyzed during the current study are available from the corresponding author on reasonable request.

Funding: None.

Conflict of interest: The authors declare that they have no conflict of interest.

References

1. Sohrabi C, Alsafi Z, O'Neill N, Khan M, Kerwan A, Al-Jabir A, et al. World Health Organization declares global emergency: A review of the 2019 novel coronavirus (COVID-19). Int J Surg. 2020;76:71-6.
2. World Health Organization. Who coronavirus disease (covid-19) dashboard 2020,November 8 [Available from: https://covid19.who.int/info.
3. Long H, Nie L, Xiang X, Li H, Zhang X, Fu X, et al. D-Dimer and Prothrombin Time Are the Significant Indicators of Severe COVID-19 and Poor Prognosis. BioMed research international. 2020;2020:6159720.
4. Guan WJ, Ni ZY, Hu Y, Liang WH, Ou CQ, He JX, et al. Clinical Characteristics of Coronavirus Disease 2019 in China. The New England journal of medicine. 2020;382(18):1708-20.

5. Aggarwal M, Dass J, Mahapatra M. Hemostatic Abnormalities in COVID-19: An Update. Indian J Hematol Blood Transfus. 2020:1-11.
6. Coccheri S. COVID-19: The crucial role of blood coagulation and fibrinolysis. Intern Emerg Med. 2020;15(8):1369-73.
7. Quintana-Díaz M, Andrés-Esteban EM, Ramírez-Cervantes KL, Olivan-Blázquez B, Juárez-Vela R, Gea-Caballero V. Coagulation Parameters: An Efficient Measure for Predicting the Prognosis and Clinical Management of Patients with COVID-19. Journal of Clinical Medicine. 2020;9(11).
8. Parasher A. COVID-19: Current understanding of its pathophysiology, clinical presentation and treatment. Postgrad Med J. 2020.
9. Al-Samkari H, Karp Leaf RS, Dzik WH, Carlson JCT, Fogerty AE, Waheed A, et al. COVID-19 and coagulation: bleeding and thrombotic manifestations of SARS-CoV-2 infection. Blood. 2020;136(4):489-500.
10. Annunziata A, Imitazione P, Polistina GE, Lanza M, Coppola A, Fiorentino G. Pulmonary Embolism in Covid-19: Coagulation Parameters, Close Monitoring to Prevent? Turk Thorac J. 2020;21(4):287-8.
11. Savioli F, Rocha LL. Coagulation profile in severe COVID-19 patients: what do we know so far? Revista Brasileira de Terapia Intensiva. 2020;32(2).
12. Buioni D, Nardi P, Ruvolo G. Thrombocytopenia and coagulation disorders due to COVID 19 infection with concomitant cardiovascular diseases requiring anti-platelet and anticoagulant therapy, which strategy? Clin Chim Acta. 2020;508:109.
13. Levi M. COVID-19 coagulopathy vs disseminated intravascular coagulation. Blood Adv. 2020;4(12):2850.
14. Quintana-Diaz M, Andres-Esteban EM, Ramirez-Cervantes KL, Olivan-Blazquez B, Juarez-Vela R, Gea-Caballero V. Coagulation Parameters: An Efficient Measure for Predicting the Prognosis and Clinical Management of Patients with COVID-19. J Clin Med. 2020;9(11).
15. WHO. COVID19. 2020. Contract No.:

.
16. Zhang Y, He L, Chen H, Lu S, Xiong Y, Liu J, et al. Manifestations of blood coagulation and its relation to clinical outcomes in severe COVID-19 patients: Retrospective analysis. Int J Lab Hematol. 2020.
17. Zhang A, Leng Y, Zhang Y, Wu K, Ji Y, Lei S, et al. Meta-analysis of coagulation parameters associated with disease severity and poor prognosis of COVID-19. International journal of infectious diseases : IJID : official publication of the International Society for Infectious Diseases. 2020;100:441-8.
18. Rauch A, Labreuche J, Lassalle F, Goutay J, Caplan M, Charbonnier L, et al. Coagulation biomarkers are independent predictors of increased oxygen requirements in COVID-19. J Thromb Haemost. 2020.
19. Luo HC, You CY, Lu SW, Fu YQ. Characteristics of coagulation alteration in patients with COVID-19. Ann Hematol. 2020.
20. Levi M, Thachil J, Iba T, Levy JH. Coagulation abnormalities and thrombosis in patients with COVID-19. Lancet Haematol. 2020;7(6):e438-e40.
21. Zou Y, Guo H, Zhang Y, Zhang Z, Liu Y, Wang J, et al. Analysis of coagulation parameters in patients with COVID-19 in Shanghai, China. Biosci Trends. 2020;14(4):285-9.

22. Sayad B, Rahimi Z. Blood coagulation parameters in patients with severe COVID-19 from Kermanshah Province, Islamic Republic of Iran. East Mediterr Health J. 2020;26(9):999-1004.
23. Adam EH, Zacharowski K, Miesbach W. A comprehensive assessment of the coagulation profile in critically ill COVID-19 patients. Thromb Res. 2020;194:42-4.
24. Paar V, Wernly B, Zhou Z, Motloch LJ, Hoppe UC, Egle A, et al. Anti-coagulation for COVID-19 treatment: both anti-thrombotic and anti-inflammatory? J Thromb Thrombolysis. 2020.
25. Belen-Apak FB, Sarialioglu F. Pulmonary intravascular coagulation in COVID-19: possible pathogenesis and recommendations on anticoagulant/thrombolytic therapy. J Thromb Thrombolysis. 2020;50(2):278-80.
26. Harenberg J, Favaloro E. COVID-19: progression of disease and intravascular coagulation - present status and future perspectives. Clin Chem Lab Med. 2020;58(7):1029-36.
27. Pizzi R, Gini G, Caiano L, Castelli B, Dotan N, Magni F, et al. Coagulation parameters and venous thromboembolism in patients with and without COVID-19 admitted to the Emergency Department for acute respiratory insufficiency. Thromb Res. 2020;196:209-12.
28. Voicu S, Delrue M, Chousterman BG, Stepanian A, Bonnin P, Malissin I, et al. Imbalance between procoagulant factors and natural coagulation inhibitors contributes to hypercoagulability in the critically ill COVID-19 patient: clinical implications. Eur Rev Med Pharmacol Sci. 2020;24(17):9161-8.
29. Mei H, Hu Y. [Characteristics, causes, diagnosis and treatment of coagulation dysfunction in patients with COVID-19]. Zhonghua Xue Ye Xue Za Zhi. 2020;41(3):185-91.
30. Bao C, Tao X, Cui W, Yi B, Pan T, Young KH, et al. SARS-CoV-2 induced thrombocytopenia as an important biomarker significantly correlated with abnormal coagulation function, increased intravascular blood clot risk and mortality in COVID-19 patients. Exp Hematol Oncol. 2020;9:16.
31. Xiong M, Liang X, Wei YD. Changes in blood coagulation in patients with severe coronavirus disease 2019 (COVID-19): a meta-analysis. Br J Haematol. 2020;189(6):1050-2.
32. Song JC, Wang G, Zhang W, Zhang Y, Li WQ, Zhou Z, et al. Chinese expert consensus on diagnosis and treatment of coagulation dysfunction in COVID-19. Mil Med Res. 2020;7(1):19.
33. Chen X, Wang Q, Xu M, Li C. A Retrospective Analysis of the Coagulation Dysfunction in COVID-19 Patients. Clin Appl Thromb Hemost. 2020;26:1076029620964868.
34. Pavoni V, Gianesello L, Pazzi M, Stera C, Meconi T, Frigieri FC. Evaluation of coagulation function by rotation thromboelastometry in critically ill patients with severe COVID-19 pneumonia. J Thromb Thrombolysis. 2020;50(2):281-6.
35. Savioli F, Rocha LL. Coagulation profile in severe COVID-19 patients: what do we know so far? Rev Bras Ter Intensiva. 2020;32(2):197-9.
36. Jose RJ, Manuel A. COVID-19 cytokine storm: the interplay between inflammation and coagulation. Lancet Respir Med. 2020;8(6):e46-e7.
37. Kasinathan G, Sathar J. Haematological manifestations, mechanisms of thrombosis and anti-coagulation in COVID-19 disease: A review. Ann Med Surg (Lond). 2020;56:173-7.
