# Supplementary material for: Blood coagulation parameter abnormalities in hospitalized patients with confirmed COVID-19 in Ethiopia
Source: PLoS One. 2021 Jun 21;16(6):e0252939. doi: 10.1371/journal.pone.0252939 (PMC8216564; doi:10.1371/journal.pone.0252939)
Supplement: Attachment — Submitted filename: PONE-D-20-35898_R2 Blood coagulation markers Ethiopia COVID.pdf [file pone.0252939.s003.pdf]

PLOS ONE

Blood coagulation parameter abnormalities in hospitalized patients with confirmed COVID-19 in Ethiopia

--Manuscript Draft--

**Manuscript Number:**	PONE-D-20-35898R2
**Article Type:**	Research Article
**Full Title:**	Blood coagulation parameter abnormalities in hospitalized patients with confirmed COVID-19 in Ethiopia
**Short Title:**	Coagulation profile of COVID-19 patients
**Corresponding Author:**	Shambel Araya<br>Addis Ababa University<br>Addis Ababa, ETHIOPIA
**Keywords:**	COVID-19; Prothrombin time; activated partial thromboplastin time; international normalized ratio; platelet; Addis Ababa; Ethiopia
**Abstract:**	Background : Coagulopathy and thromboembolic events are among the complications of Corona Virus disease 2019 (COVID-19). Thus, abnormal coagulation profiles in COVID-19 patients are taken as important prognostic factors of COVID-19 disease severity. The aim of this study was to analyze coagulation profiles of hospitalized COVID-19 patients in Addis Ababa, Ethiopia. Methods : This cross-sectional study was conducted among 455 Covid-19 patients admitted at Millennium COVID-19 treatment center, Addis Ababa, Ethiopia from July 1- October 23, 2020. Prothrombin Time (PT), Activated Partial Thromboplastin Time (APTT) and International normalized ratio (INR) were determined on HUMACLOT DUE PLUS coagulation analyzer (Wiesbaden, Germany) . In all tests, p<0.05 was defined as statistically significant.<br>Result : A prolonged prothrombin time was found in 46.8% of study subjects with COVID-19 and a prolonged prothrombin time and elevated INR with 53.3% of study subjects with severe and 51 % of critically COVID patients. Thrombocytopenia was detected in 22.1% of COVID-19 patients. 50.5% and 51.3% of COVID-19 patients older than 55 years had thrombocytopenia and prolonged APTT respectively.<br>Conclusion :<br>In this study, prolonged prothrombin time and high INR were detected in around 50% of severe and critical COVID-19 patients. Thrombocytopenia and prolonged APTT were dominant in COVID-19 patients older than 55 years. Thus, we recommend emphasis to be given for monitoring of platelet count, PT, APTT and INR in hospitalized COVID-19 patients.
**Order of Authors:**	Shambel Araya, MSc
	Mintesnot Aragaw Mamo
	Yakob Gebregziabher Tsegay
	Aschalew Aytenew
	Abebe Hordofa
	AbebeEdao Negeso
	Zemenu Tamir
	Tirhas Niguse
	Mahlet Cheru
	Asegdew Atlaw
	Moges Wordofa
**Response to Reviewers:**	Cover letter<br>Shambel Araya<br>Addis Ababa University<br>Addis Ababa, Ethiopia

Powered by Editorial Manager® and ProduXion Manager® from Aries Systems Corporation

Email: shambelaraya8@gmail.com

Date: April, 2021
To: PLOS ONE Journal
Dear Editorial:
We are glad to write this response that our research article entitled “Blood coagulation parameter abnormalities among patients with confirmed COVID-19 in Ethiopia” (Submission ID: PONE-D-20-35898R1) has been requested to review for publication in PLOS ONE journal. We are pleased to have an opportunity to make our paper revised and we have greatly appreciated the reviewers’ and editor’s comments and suggestions were very helpful overall. In revising the paper, we have carefully considered reviewers’ and editor’s comments and suggestions on our revised submission. As instructed, we have attempted to succinctly explain changes made in reaction to all comments and reply to each comment in point-by-point fashion as follows:

Response to comments and suggestions inserted in the PDF format of the Manuscript

Comment 1: Abstract, methods section: “...Estimated by...” was replaced by “...determined on...” as per your suggestion in line 34
Comment 2: Abstract, Result section: “among” is replaced with ‘in’ in line 36
Comment 3: Abstract, Result section: “…Prolonged prothrombin time and high INR were seen among 53.3% severe and 51% critical 38 patients with COVID-19 manifestation…. Thrombocytopenia was detected in 22.1% of COVID-19 39 patients” has replaced with… and a prolonged prothrombin time and elevated INR with 53.3% of study subjects with severe and 51 % of critically COVID patients…” in line 37-39
Comment 4: abstract, conclusion section. In this study, prolonged prothrombin time and high INR were found among severe and critical COVID-19 patients.
Revised: In this study, prolonged prothrombin time and high INR were detected in 50% of severe and critical COVID-19 patients
Comment: abstract, conclusion section. Delete management
Response: Accepted and deleted in line 45
Comment 6: Introduction section. Replace showed with demonstrated that
Response: Accepted and replaced
Comment 7: …Introduction section. coagulation parameters could be an efficient measure for improving the clinical management and predicting the prognosis of patients with SARS COV-2(7)…
Revised: …coagulation parameters could be an efficient measure for predicting the prognosis of patients with SARS COV-2(7) and guiding management…
Comment 8: Introduction section: Accepted and modified as follow
“Different studies also support that COVID-19 patients are at high risk of developing disseminated intravascular coagulation (12, 13). It is also indicated that comparison of reports from various populations related to the clinical course, outcome of COVID-19 and blood coagulation profile in these patients are necessary to help the management and treatment of the disease (12, 14). Moreover, this routine coagulation parameter tests could be used as potential indicators for COVID-19 in individuals having typical clinical manifestations that would be inputs for prompt patient management especially in resource limited settings where the high-tech gold standard RT-PCR is not widely available, like Ethiopia”
Revised: Several studies have also demonstrated the increased occurrence of intravascular disseminated coagulopathy (DIC) in patients with COVID-19 (12, 13). The result of blood coagulation parameters in COVID-19 can also guide management decisions and improve outcomes (12, 14).
Moreover, routine coagulation parameter tests results could potentially be utilized in symptomatic patients in resource limited settings with inadequate access to COVID-19 RT-PCR, as in Ethiopia, to raise suspension of this infection. However, data on coagulation profiles among Ethiopian COVID-19 patients is not readily available, like

	Ethiopia.<br>Comment8: Introduction section Replace "…find out…" with "...Determine…"<br>Response: accepted and replaced as per your suggestion<br>Comment 9: Delete "and" in line 88<br>Line 92: Replace "…of…" with "…for…"<br>Line 94: Replace "…Was taking…" with "…were receiving…"<br>Line 95: Change "According to" in to "…with…"<br>Response: Accepted and modified accordingly<br>Comment 10: Methods, laboratory section.<br>Eight milliliters of venous blood were collected by professional nurses working in the treatment center: five milliliters in EDTA for platelet count, three milliliters in 3.2% sodium citrated<br>Revised: Venous bloods were collected by professional nurses working in the treatment center: 5 mL in EDTA and 3 mL in 3.2% sodium citrated<br>Comment 11: Insert trade mark for each instruments and software's<br>Response: accepted and inserted as follow in line 99-102<br>Comment12: remove Addis Ababa, Ethiopia, 2020 from table 1<br>Response: Deleted as per your suggestion<br>Comment 13: Result section line 120-122 "Severe and critical groups showed differences in sex ratio and age distribution. In severe and critical groups, majority were males and elderly (Table 2)."<br>Revised: Severe and critical groups showed differences in sex ratio and age distribution. In severe (36.6%) and critical groups (48.5%), were elderly males of the age of >55 years old. y (Table 2).<br>Comment 14: result section table 3: remove "…admitted to Millennium COVID-19 treatment center Addis Ababa, Ethiopia…"<br>Response: accepted and removed table heading<br>Comment 15: Result section line 127-129, rewrite "Prolonged PT value was found among males (49.8%) than females (41%) and it has a significant association with gender (P = 0.045)."<br>Revised: accepted and modified as "Prolonged PT values were demonstrated more frequently among males (49.8%) than females (41%) and this difference was significantly different (P = 0.045)."<br>Comment 16: replace study subjects with individuals<br>Response accepted and replaced in line 130<br>Comment 16 result section line 135-136: rearrange "…patients aged older than 55 years had thrombocytopenia. Thrombocytopenia was higher among…" in to<br>Revised: modified as "…patients aged older than 55 years had thrombocytopenia and the occurrence was higher among…"<br>Comment 17: insert "…Result of …" in table 3 heading<br>Response: accepted and modified as follow "Result of coagulation parameters in patients with severe COVID-19 according to different variables"<br>Comment 18: Discussion section: Replace "…had brought…" with "...had a…"<br>Delete indicated as<br>Remove findings<br>Comment 19: Rearrange the paragraph as below for "Coagulation abnormalities are indicated as frequent findings in COVID-19 patients and associated with poor prognosis and survival(7). Similarly, it is also indicated that coagulopathy which is resulted due to dysregulation of coagulation and associated with hypercoagulability as evidenced by venous and arterial thrombosis and multiorgan dysfunction (16); is one of the most significant prognostic factors in patients with COVID-19 and is associated with increased hospitalization, admission to critical care, and mortality"<br>Response: accepted<br>Revised: Coagulation abnormalities are frequent in COVID-19 patients and are associated with poor prognosis and reduced survival(7). Dysregulation of coagulation and associated with hypercoagulability in patients with COVID manifest as venous and arterial thrombosis and multiorgan dysfunction (16); which are poor prognostic markers resulting in increased mortality and hospitalization and ICU admission<br>Comment 20: discussion section<br>Line 151: remove "…levels..."<br>Line 153 insert "…more frequent…"<br>Line 153: remove "…than moderate ones…"<br>Line 159: replace "… alongside these, it is suggested that…" with "and"<br>Line 164: replace "…Evidence…" with "…published studies…"

Powered by Editorial Manager® and ProduXion Manager® from Aries Systems Corporation

	Response: Accepted and modified as per your suggestion<br>Line 167: replace "…clotting is reported from…" with "…Abnormal thrombosis of…"<br>Line 167-170: rewrite it as follow for "…Clotting is reported from different medical devices used, deep vein thrombosis and multiple thrombi in the vessels of the lungs, kidneys and other organs from autopsy of patients died of Covid-19 (9, 29). These indicate clinicians to use…"<br>Response: Accepted and rephrase as follow<br>Revised: "…Abnormal thrombosis of different medical devices, deep vein thrombosis and multiple thrombi in the vessels of the lungs, kidneys and other organs at autopsy of patients who died of Covid-19 have been reported serving as the impetus behind guidelines (9, 29)which support the use…"<br>Line 173-175: rewrite it as follow for "Studies across suggested that routine coagulation tests can be considered as a significant marker to help clinicians assess prognosis and severity of patients with COVID-19…"<br>Response: Accepted and rephrase as follow<br>Revised: (34). "...Studies suggest that routine coagulation test results are markers of disease severity and assist in management decision…"<br>Line 184: Insert "… in critically ill non-COVID patients…"<br>Response: accepted<br>Line 186-189: rephrase the sentence "COVID-19-associated coagulopathy is however unique with a much-decreased platelet count (9, 36-38)"<br>Revised: Published studies support that COVID-19-associated coagulopathy is characterized by a decreased platelet count (9, 36-38).<br>Dear all, we are very grateful for your valuable comments and for your time<br>Looking forward to hearing from you. Thank you again for your consideration!<br>Sincerely,<br>Shambel Araya (BSc, MSc, PhD fellow)
**Additional Information:**	
**Question**	**Response**
**Financial Disclosure**<br><br>Enter a financial disclosure statement that describes the sources of funding for the work included in this submission. Review the submission guidelines for detailed requirements. View published research articles from *PLOS ONE* for specific examples.<br><br>This statement is required for submission and **will appear in the published article** if the submission is accepted. Please make sure it is accurate.	The author(s) received no specific funding for this work.

Powered by Editorial Manager® and ProduXion Manager® from Aries Systems Corporation

**Unfunded studies**<br>Enter: *The author(s) received no specific funding for this work.*<br><br>**Funded studies**<br>Enter a statement with the following details:<br>• Initials of the authors who received each award<br>• Grant numbers awarded to each author<br>• The full name of each funder<br>• URL of each funder website<br>• Did the sponsors or funders play any role in the study design, data collection and analysis, decision to publish, or preparation of the manuscript?<br>• **NO** - Include this sentence at the end of your statement: *The funders had no role in study design, data collection and analysis, decision to publish, or preparation of the manuscript.*<br>• **YES** - Specify the role(s) played.<br><br>* typeset	
**Competing Interests**<br><br>Use the instructions below to enter a competing interest statement for this submission. On behalf of all authors, disclose any competing interests that could be perceived to bias this work—acknowledging all financial support and any other relevant financial or non-financial competing interests.<br><br>This statement **will appear in the published article** if the submission is accepted. Please make sure it is accurate. View published research articles from *PLOS ONE* for specific examples.	The authors have declared that no competing interests exist

**NO authors have competing interests**<br><br>Enter: *The authors have declared that no competing interests exist.*<br><br>**Authors with competing interests**<br><br>Enter competing interest details beginning with this statement:<br><br>*I have read the journal's policy and the authors of this manuscript have the following competing interests: [insert competing interests here]*<br><br>* typeset	
**Ethics Statement**<br><br>Enter an ethics statement for this submission. This statement is required if the study involved:<br><br>• Human participants<br>• Human specimens or tissue<br>• Vertebrate animals or cephalopods<br>• Vertebrate embryos or tissues<br>• Field research<br><br>Write "N/A" if the submission does not require an ethics statement.<br><br>General guidance is provided below. Consult the submission guidelines for detailed instructions. **Make sure that all information entered here is included in the Methods section of the manuscript.**	Addis Ababa University College of Health Science Department of Medical Laboratory Science research ethics review committee (DRERC) protocol number: DRERC/538/20/MLS

Powered by Editorial Manager® and ProduXion Manager® from Aries Systems Corporation

**Format for specific study types**<br><br>**Human Subject Research (involving human participants and/or tissue)**<br>• Give the name of the institutional review board or ethics committee that approved the study<br>• Include the approval number and/or a statement indicating approval of this research<br>• Indicate the form of consent obtained (written/oral) or the reason that consent was not obtained (e.g. the data were analyzed anonymously)<br><br>**Animal Research (involving vertebrate animals, embryos or tissues)**<br>• Provide the name of the Institutional Animal Care and Use Committee (IACUC) or other relevant ethics board that reviewed the study protocol, and indicate whether they approved this research or granted a formal waiver of ethical approval<br>• Include an approval number if one was obtained<br>• If the study involved *non-human primates*, add *additional details* about animal welfare and steps taken to ameliorate suffering<br>• If anesthesia, euthanasia, or any kind of animal sacrifice is part of the study, include briefly which substances and/or methods were applied<br><br>**Field Research**<br><br>Include the following details if this study involves the collection of plant, animal, or other materials from a natural setting:<br>• Field permit number<br>• Name of the institution or relevant body that granted permission	
**Data Availability**<br><br>Authors are required to make all data underlying the findings described fully available, without restriction, and from the time of publication. PLOS allows rare exceptions to address legal and ethical concerns. See the PLOS Data Policy and FAQ for detailed information.	Yes - all data are fully available without restriction

A Data Availability Statement describing where the data can be found is required at submission. Your answers to this question constitute the Data Availability Statement and **will be published in the article**, if accepted.<br><br>**Important:** Stating 'data available on request from the author' is not sufficient. If your data are only available upon request, select 'No' for the first question and explain your exceptional situation in the text box.<br><br>Do the authors confirm that all data underlying the findings described in their manuscript are fully available without restriction?	
**Describe where the data may be found in full sentences. If you are copying our sample text, replace any instances of XXX with the appropriate details.**<br><br>• If the data are **held or will be held in a public repository**, include URLs, accession numbers or DOIs. If this information will only be available after acceptance, indicate this by ticking the box below. For example: *All XXX files are available from the XXX database (accession number(s) XXX, XXX.*).<br>• If the data are all contained **within the manuscript and/or Supporting Information files**, enter the following: *All relevant data are within the manuscript and its Supporting Information files.*<br>• If neither of these applies but you are able to provide **details of access elsewhere**, with or without limitations, please do so. For example:<br><br>*Data cannot be shared publicly because of [XXX]. Data are available from the XXX Institutional Data Access / Ethics Committee (contact via XXX) for researchers who meet the criteria for access to confidential data.*<br><br>*The data underlying the results presented in the study are available from (include the name of the third party*	All relevant data are within the manuscript

and contact information or URL).<br>• This text is appropriate if the data are owned by a third party and authors do not have permission to share the data.<br><br>* typeset	
Additional data availability information:	

Manuscript Click here to access/download;Manuscript;Manuscript 02042021.docx

Blood coagulation parameter abnormalities in hospitalized patients with confirmed COVID-19 in Ethiopia

Shambel Araya[1&2*], MintesnotAragaw Mamo [1&2], YakobGebregziabher Tsegay[3&4], Asegdew Atlaw[2], Aschalew Aytenew[2], Abebe Hordofa[2], Abebe Edao Negeso[1], MogesWordofa[1], Tirhas Niguse[1], Mahlet Cheru[1],Zemenu Tamir[1]

[1]Department of Medical Laboratory Sciences, College of Health Sciences, Addis Ababa University, Addis Ababa, Ethiopia

[2]Department of Medical Laboratory, Millennium COVID-19 Treatment and Care Centre, St. Paual Millennium Medical College, Addis Ababa, Ethiopia;

[3]Department of Medical Biotechnology, Institute of Biotechnology, University of Gondar, Gondar, Ethiopia.

[4]Department of Research and development center, College of Health Sciences, Defense University, Addis Ababa, Ethiopia.

Address:

1. Shambel Araya (corresponding author);shambelaraya8@gmail.com
2. MintsnotAragaw Mamo:mintsh2015@gmail.com
3. YakobGebreegziabherTsegaye: yakobtsegay17@gmail.com
4. AsegdewAtlaw: asegdew21@gmail.com
5. AschalewAytenew:aschu9033@gmail.com
6. Abebe Hordofa: abuhordofa@gmail.com
7. Abebe EdaoNegeso:abenegesso@gmail.com
8. MogesWordofa: heranmakmow@gmail.com
9. TirhasNiguse;peace.for.all.060610@gmail.com
10. MahletCheru:yuluyaya54@gmail.com
11. Zemenu Tamir: zemenut266@gmail.com

Abstract

Background: Coagulopathy and thromboembolic events are among the complications of Corona Virus disease 2019 (COVID-19). Thus, abnormal coagulation profiles in COVID-19 patients are taken as important prognostic factors of COVID-19 disease severity. The aim of this study was to analyze coagulation profiles of hospitalized COVID-19 patients in Addis Ababa, Ethiopia. **Methods**: This cross-sectional study was conducted among 455 Covid-19 patients admitted at Millennium COVID-19 treatment center, Addis Ababa, Ethiopia from July 1- October 23, 2020. Prothrombin Time (PT), Activated Partial Thromboplastin Time (APTT) and International normalized ratio (INR) were determined on HUMACLOT DUE PLUS coagulation analyzer (Wiesbaden, Germany). In all tests, $p<0.05$ was defined as statistically significant.

Result: A prolonged prothrombin time was found in 46.8% of study subjects with COVID-19 and a prolonged prothrombin time and elevated INR with 53.3% of study subjects with severe and 51 % of critically COVID patients. Thrombocytopenia was detected in 22.1% of COVID-19 patients. 50.5% and 51.3% of COVID-19 patients older than 55 years had thrombocytopenia and prolonged APTT respectively.

Conclusion:
In this study, prolonged prothrombin time and high INR were detected in around 50% of severe and critical COVID-19 patients. Thrombocytopenia and prolonged APTT were dominant in COVID-19 patients older than 55 years. Thus, we recommend emphasis to be given for monitoring of platelet count, PT, APTT and INR in hospitalized COVID-19 patients.

Key words: COVID-19, Prothrombin time, activated partial thromboplastin time, international normalized ratio, Platelet, Addis Ababa, Ethiopia.

Introduction

Coronavirus disease 2019 (COVID-19) is caused by a novel beta corona virus called severe acute respiratory syndrome coronavirus 2 (SARS-CoV-2)(1). COVID-19 has become a pandemic that has heavily affected the global population. As of November 8, 2020, there have been 49,578,590 confirmed cases of COVID-19 and 1,245,717 deaths, reported to World Health organization (WHO). Similarly, there have been 99,204 confirmed cases of COVID-19 with 1,518 deaths in Ethiopia(2).

The severity of COVID-19 varies considerably from asymptomatic to life threatening, lung injury being the main clinical manifestation. Most of the patients have a favorable prognosis, but some rapidly progress to severe and critical cases with respiratory distress syndrome, coagulation dysfunction and multiple organ failures (3, 4). Although the pathophysiology and the underlining mechanisms of clinical manifestations remain unclear, thrombo-inflammation and cytokine storm are clearly established components in Severe Acute Respiratory Distress Syndrome (SARS) of COVID-19(5-8).

Coagulopathy and abnormal coagulation profiles were indicated among the most significant markers of poor prognosis in COVID-19 patients (9-11). A retrospective cohort study conducted in Spain Madrid demonstrated that COVID-19 non-survivors had significantly lower prothrombin activity, abnormal coagulation parameters like prolonged PT, APTT, higher D-dimer and higher fibrinogen levels compared to survivors indicating coagulation parameters could be an efficient measure for predicting the prognosis of patients with SARS COV-2(7) and guiding management. Similarly, Long et al has reported that occurrence of coagulation dysfunction is more likely in severe and critically ill patients. The study also showed that D-dimer and prothrombin time could be considered as main indicators in predicting the mortality of

COVID-19 patients (3). Several studies have also demonstrated the increased occurrence of intravascular disseminated coagulopathy (DIC) in patients with COVID-19 (12, 13). The result of blood coagulation parameters in COVID-19 can also guide management decisions and improve outcomes (12, 14).

Moreover, routine coagulation parameter tests results could potentially be utilized in symptomatic patients in resource limited settings with inadequate access to COVID-19 RT-PCR, as in Ethiopia, to raise suspension of this infection. However, data on coagulation profiles among Ethiopian COVID-19 patients is not readily available. Thus, the aim of this study was to determinethe coagulation profile of COVID-19 patients admitted at Millennium COVID-19 treatment center, Addis Ababa, Ethiopia.

Methods

Ethical consideration: The study was approved by Addis Ababa University College of Health Sciences, department of Medical Laboratory Sciences research ethics review committee (DRERC/538/20/MLS) and it was in accordance with the principles of the Helsinki II declaration. Laboratory test results were communicated to the responsible clinicians working at the treatment center. Written informed consent was obtained from the study participants. All the information obtained from the study participants were kept confidential.

Study population

In this study, we have included 455 consecutive patients with confirmed SARS-CoV-2 infection admitted to Millennium COVID-19 treatment center, Addis Ababa, Ethiopia from July 1-October 23, 2020. The treatment center is the first referral center for COVID-19 patients in Ethiopia, since May 02, 2020. None of the study participants were receving anticoagulant

medications before blood drawing. Diagnosis of SARS-CoV-2 infection was made with real time PCR.

Sample collection and coagulation profile analysis

2.6. Laboratory Analysis

Venous bloods were collected by professional nurses working in the treatment center: 5 mL in EDTA and 3 mL in 3.2% sodium citrated anti-coagulated tube for analysis of coagulation parameters. The samples for coagulation tests were collected at hospital admission. The prothrombin time (PT), activated partial prothrombin time (APTT), and international normalized ratio (INR) were analyzed using HUMACLOT DUE PLUS® coagulation analyzer (Wiesbaden ®, Germany). Platelet count was performed using UniCel® DxH 800 Coulter®Cellular Analysis System (Beckman Coulter ®, Inc. 4300 N. Harbor Blvd. Fullerton, CA 92835). The coagulation parameters were compared with the manufacturer cut off normal range of PT = 11.7- 15 seconds, APTT = 23.8- 37.9 seconds, INR = 1.0- 1.2 and PLT= 159-386/µ.l. The coagulation parameters above the cut off range were considered as a prolonged ~~time~~ and thrombocytopenia in the case of lower than cut off value for platelet. All laboratory tests and ~~its~~ interpretation were done following the manufacturers' recommendation and standard operating procedures.

Statistical Analysis

Statistical Package for the Social Sciences (SPSS) software version 25.0 (SPSS® Inc., Chicago, IL, USA) was used for statistical analysis. Chi-square test was used to determine association among categorical variables. The quantitative data were expressed as Mean ± SD. P value < 0.05 was considered as statistically significant.

Results

Socio-demographic and Clinical characteristics of Study participants

In this study, 455 patients diagnosed with COVID-19 were included. Among the study participants, 289 (63.5%) were males. The study participants were between the age of 4 and 90 years with a mean of 49.9 ±18.3 years. From the total 455 case, there were 297 mild cases, 90 severe cases, and 68 critical cases based on disease severity of COVID-19(Table 1).

Table 1: Socio-demographic characteristics of study participants

Variables		Frequency	Percent
Gender	Male	289	63.5%
	Female	166	36.5%
Age group	0-18 years	15	3.2%
	18-35 years	101	22.1%
	36-55 years	158	34.7%
	>55 years	181	39.7%
Disease severity	Moderate	297	65.2%
	Severe	90	19.8%
	Critical	68	15%

The median time from the disease onset to admission was 4 days (2-8 days). Severe and critical groups showed differences in sex ratio and age distribution. In severe (36.6%) and critical groups (48.5%), were elderly males of the age of >55 years old. ~~y~~ (Table 2).

Table 2: Socio-demographic characteristics and disease severity of COVID-19 patients

Variables		Disease Severity			P-value
		Moderate, n (%)	Severe, n (%)	Critical, n (%)	
Age(year)	0-18, n= 15	10(66.7)	4(26.7)	1(6.7)	0.283
	18-35, n=101	65(64.35)	22(21.78)	14(13.8)	
	36-55, n=158	107(67.7)	31(19.6)	20(12.65)	
	>55, n=181	115(63.5)	33(18.2)	33(8.2)	
Sex, ~~N=455~~	Male, n=289	187(64.7)	56(19.3)	46(15.9)	0.045
	Female, n=166	110(66.2)	34(20.4)	22(13.2)	

Magnitude of coagulation abnormalities

In this study, 209 COVID-19 patients (46%) showed prolonged PT and ~~higher~~ INR values. Among those patients with prolonged PT, 51.3% were above 55 years of age. Prolonged PT values were demonstrated more frequently among males (49.8%) than females (41%) and this difference was statistically different(P = 0.045). Similarly, 51.4% and 53.3% of ICU (critical) and severe patients had prolonged PT values. Notably, prolonged APTT values were found among 43.1% of individuals, and from these 47%, 45% and 41% were among ICU (critical), severe and moderate patients, respectively. 57.2% of female patients had prolonged APTT; and 51.3% of patients aged older than 55 years had a prolonged APTT.

Thrombocytopenia was detected in 22.1% (101 out of 455) study subjects. 50.5% (50 out of 99) patients aged older than 55 years had thrombocytopenia and the occurrence was higher among male (23.8%) than female (18%) ICU patients (Table 3).

Coagulation Parameters		***Variables***								
		Age				***Sex***		***Disease Severity***		
		0-18 n(%)	***19-35 n(%)***	***36-55 n(%)***	***>55 n(%)***	***Male n(%)***	***Female n(%)***	***Moderate n(%)***	***Severe n(%)***	***Critical n(%)***
PT	High n=213	9(4.2)	50(23.47)	61(28.6	93(43.6)	144(67.6)	69(32.4)	130(61)	48(22.5)	35(16.4)
	Normal n=220	6(2.7)	45(20.45)	89(40.4)	80(36.3)	131(59.5)	89(40.4)	149(67.7)	40(18.1)	31(14.1)
	Low=22	0	6(27.2)	8(36.3)	8(36.3)	14(63.6)	8(36.3)	18(81)	2(9)	2(9)
APTT	High=196	6(3)	46(23.4)	68(34.7)	76(38.77)	101(51.5)	95(48.5)	115(58.67)	41(21)	42(21.4)
	Normal n=193	6(3.1)	38(19.7)	70(36.2)	79(41)	136(70.4)	57(29.5)	137(71)	36(18.6)	21(10.8)
	Low n=66	3(4.5)	17(25.7)	20(30.3)	26(39.3)	52(78.7)	14(21)	45(68)	13(19.7)	5(7.5)
PLT	High n=65	4(6.1)	11(17)	24(37)	26(40)	43(66)	22(33.8)	39(60)	8(12.3)	8(12.3)
	Normal n=289	8(3)	70(24.2)	105(36.3)	105(36.3)	175(60.8)	114(39)	214(74)	44(15)	31(10.6)
	Low n= 101	3(2.9)	20(20)	28(27.7)	50(49.5)	69(69.70	30(30.3)	33(32)	38(37.6)	30(29.7)
INR	High n=210	9(4.2)	50(24.7)	60(28.5)	91(43.3)	141(67)	69(32.8)	127(60.4)	50(23.8)	33(15.7)

	Normal =224	5(2.2)	44(19.6)	93(41.5)	82(36.6)	115(51)	75(33.4)	113(50.4)	45(20)	32(14.2)
	Low n=21	1(4.7)	7(33.3)	5(23.8)	8(38)	14(66.6)	7(33.3)	15(71)	3(14.5)	3(14.5)

Table 3: Result of coagulation parameters in patients with severe COVID-19 according to different variables

PLT=platelet; PT= prothrombin time; APTT=activated partial thromboplastin time; INR=international normalized ratio.

Discussion

The COVID-19 pandemic had a major impact on health care globally. COVID-19 has already caused >1.2 million deaths worldwide and more than 1400 in Ethiopia as of October 30,2020 according to WHO report(15). Coagulation abnormalities are frequent in COVID-19 patients and are associated with poor prognosis and reduced survival(7). The dysregulation of coagulation ~~and~~ associated with hypercoagulability ~~in patients with COVID~~ manifest as venous and arterial thrombosis and multiorgan dysfunction(16)~~;~~ which are poor prognostic markers ~~resulting in increased mortality and hospitalization and ICU admission~~ (13, 14, 17-19). Previous studies indicated that coagulopathy in patients hospitalized with COVID-19 is characterized by ~~increase~~ in coagulation parameters such as PT, APTT and INR (20, 21).

Patients with serious infection are more likely to have COVID-19 associated coagulopathy than patients with a mild infection (21,22). In our study, prolonged PT, APTT ~~an~~ INR ~~was~~ more frequent among severe and critical COVID-19 patients. Similarly, studies also reported that thrombotic complications are common among COVID-19 patients admitted to intensive care unit (ICU) ~~(9.5%-47%)~~(22-24).

Treatment of the underlying condition is suggested to be paramount in coagulopathies. It is shown that bleeding is not common clinical manifestation in COVID-19 infections despite abnormal coagulation parameters (23,24). and supportive care including blood product transfusion should be individualized in COVID -19 patients(25, 26). Laboratory findings alone should not be taken as basis for instituting blood transfusion therapy, rather it should be reserved for those who are bleeding, requires an invasive procedure, or who are otherwise at high risk for bleeding complications (26, 27).

Published studies indicate that COVID-19 is associated with a hyper-coagulable state. Venous thromboembolism (VTE) and arterial thrombosis ranging from 15% to 30% were found in critically ill patients with COVID-19 and about 7% in those admitted to medical wards (28-30). Abnormal thrombosis of different medical devices, deep vein thrombosis and multiple thrombi in the vessels of the lungs, kidneys and other organs at autopsy of patients who died of Covid-19 have been reported serving as the impetus behind guidelines (9, 29)which support the use of therapeutic doses of heparin or low-molecular-weight heparin instead of prophylactic doses in critically ill COVID-19 patients (12, 26, 31).In the current study, thrombocytopenia was observed among males (23.8%) than females (19.8%) and older people (27.6%). Severe (42.68%) and critical (42%) patients also had thrombocytopenia and this was in line with studies conducted in different countries (20, 22, 32, 33). Thrombocytopenia, defined as platelet count less than 100×10^9 cells/L were independently associated with COVID-19 severity(34). Studies suggest that routine coagulation test results are markers of disease severity and assist in management decision.In critically ill patients, thrombocytopenia correlates with multi-organ failure and death, and a decline in platelet count, even in the absence of overt thrombocytopenia, portends a worse outcome (9, 12, 13). In patients who are not bleeding, there is no evidence that

correction of laboratory parameters with blood products improves outcomes. Replacement might worsen disseminated thrombosis and further deplete scarce blood products (28, 35).

Many studies reported that coagulopathy associated with COVID-19 is characterized by thrombocytopenia, prolongation of the prothrombin time, high levels of D-dimer, and elevated levels of fibrinogen, factor VIII, and von Willebrand factor (3, 11, 16). ~~The degree of coagulation abnormalities in critically ill non-COVID patients correlates with disease severity and predict the risk of thrombosis, the need for ventilator support, and mortality.~~ Published studies ~~support~~ that COVID-19-associated coagulopathy is characterized by a decreased platelet count (9, 36-38)~~.~~ ~~Patients with critical COVID-19 infection~~ and a cytokine storm ~~have~~ an extreme hyper-coagulable state. Even though the reason for this life-threatening condition is not known, this might be due to an uncontrolled hyper-inflammatory response without previous immunity (39, 40).

Conclusion: In this study, prolonged prothrombin time and high INR were found among severe and critical COVID-19 patients. Thrombocytopenia and prolonged ~~APTT~~ were dominant in COVID-19 patients older than 55 years. Thus, we recommend emphasis to be given for monitoring of platelet count, PT, APTT and INR in hospitalized COVID-19 patients management.

Consent for publication: Not applicable

Availability of data and material

All the available data were included in the manuscript.

Funding: None

Conflictof interest: The authors declare that they have no conflict of interest.

References

1. Sohrabi C, Alsafi Z, O'Neill N, Khan M, Kerwan A, Al-Jabir A, et al. World Health Organization declares global emergency: A review of the 2019 novel coronavirus (COVID-19). Int J Surg. 2020;76:71-6.
2. World Health Organization. Who coronavirus disease (covid-19) dashboard 2020,November 8 [Available from: https://covid19.who.int/info.
3. Long H, Nie L, Xiang X, Li H, Zhang X, Fu X, et al. D-Dimer and Prothrombin Time Are the Significant Indicators of Severe COVID-19 and Poor Prognosis. BioMed research international. 2020;2020:6159720.
4. Guan WJ, Ni ZY, Hu Y, Liang WH, Ou CQ, He JX, et al. Clinical Characteristics of Coronavirus Disease 2019 in China. The New England journal of medicine. 2020;382(18):1708-20.
5. Aggarwal M, Dass J, Mahapatra M. Hemostatic Abnormalities in COVID-19: An Update. Indian J Hematol Blood Transfus. 2020:1-11.
6. Coccheri S. COVID-19: The crucial role of blood coagulation and fibrinolysis. Intern Emerg Med. 2020;15(8):1369-73.
7. Quintana-Díaz M, Andrés-Esteban EM, Ramírez-Cervantes KL, Olivan-Blázquez B, Juárez-Vela R, Gea-Caballero V. Coagulation Parameters: An Efficient Measure for Predicting the Prognosis and Clinical Management of Patients with COVID-19. Journal of Clinical Medicine. 2020;9(11).
8. Parasher A. COVID-19: Current understanding of its pathophysiology, clinical presentation and treatment. Postgrad Med J. 2020.
9. Al-Samkari H, Karp Leaf RS, Dzik WH, Carlson JCT, Fogerty AE, Waheed A, et al. COVID-19 and coagulation: bleeding and thrombotic manifestations of SARS-CoV-2 infection. Blood. 2020;136(4):489-500.
10. Annunziata A, Imitazione P, Polistina GE, Lanza M, Coppola A, Fiorentino G. Pulmonary Embolism in Covid-19: Coagulation Parameters, Close Monitoring to Prevent? Turk Thorac J. 2020;21(4):287-8.
11. Savioli F, Rocha LL. Coagulation profile in severe COVID-19 patients: what do we know so far? Revista Brasileira de Terapia Intensiva. 2020;32(2).
12. Buioni D, Nardi P, Ruvolo G. Thrombocytopenia and coagulation disorders due to COVID 19 infection with concomitant cardiovascular diseases requiring anti-platelet and anticoagulant therapy, which strategy? Clin Chim Acta. 2020;508:109.
13. Levi M. COVID-19 coagulopathy vs disseminated intravascular coagulation. Blood Adv. 2020;4(12):2850.
14. Quintana-Diaz M, Andres-Esteban EM, Ramirez-Cervantes KL, Olivan-Blazquez B, Juarez-Vela R, Gea-Caballero V. Coagulation Parameters: An Efficient Measure for Predicting the Prognosis and Clinical Management of Patients with COVID-19. J Clin Med. 2020;9(11).
15. WHO. COVID19. 2020. Contract No.:

.
16. Chan NC, Weitz JI. COVID-19 coagulopathy, thrombosis, and bleeding. Blood. 2020;41:100648.

17. Rauch A, Labreuche J, Lassalle F, Goutay J, Caplan M, Charbonnier L, et al. Coagulation biomarkers are independent predictors of increased oxygen requirements in COVID-19. J Thromb Haemost. 2020.

18. Zhang Y, He L, Chen H, Lu S, Xiong Y, Liu J, et al. Manifestations of blood coagulation and its relation to clinical outcomes in severe COVID-19 patients: Retrospective analysis. Int J Lab Hematol. 2020.

19. Zhang A, Leng Y, Zhang Y, Wu K, Ji Y, Lei S, et al. Meta-analysis of coagulation parameters associated with disease severity and poor prognosis of COVID-19. International journal of infectious diseases : IJID : official publication of the International Society for Infectious Diseases. 2020;100:441-8.

20. Luo HC, You CY, Lu SW, Fu YQ. Characteristics of coagulation alteration in patients with COVID-19. Ann Hematol. 2020.

21. Levi M, Thachil J, Iba T, Levy JH. Coagulation abnormalities and thrombosis in patients with COVID-19. Lancet Haematol. 2020;7(6):e438-e40.

22. Zou Y, Guo H, Zhang Y, Zhang Z, Liu Y, Wang J, et al. Analysis of coagulation parameters in patients with COVID-19 in Shanghai, China. Biosci Trends. 2020;14(4):285-9.

23. Sayad B, Rahimi Z. Blood coagulation parameters in patients with severe COVID-19 from Kermanshah Province, Islamic Republic of Iran. East Mediterr Health J. 2020;26(9):999-1004.

24. Adam EH, Zacharowski K, Miesbach W. A comprehensive assessment of the coagulation profile in critically ill COVID-19 patients. Thromb Res. 2020;194:42-4.

25. Paar V, Wernly B, Zhou Z, Motloch LJ, Hoppe UC, Egle A, et al. Anti-coagulation for COVID-19 treatment: both anti-thrombotic and anti-inflammatory? J Thromb Thrombolysis. 2020.

26. Belen-Apak FB, Sarialioglu F. Pulmonary intravascular coagulation in COVID-19: possible pathogenesis and recommendations on anticoagulant/thrombolytic therapy. J Thromb Thrombolysis. 2020;50(2):278-80.

27. Lee AY, Connors JM, Kreuziger LB, Murphy M, Gernsheimer T, Lin Y, et al. COVID-19 and Coagulopathy 2020, December 1 [Available from: https://www.hematology.org/COVID-19/COVID-19-and-coagulopathy.

28. Harenberg J, Favaloro E. COVID-19: progression of disease and intravascular coagulation - present status and future perspectives. Clin Chem Lab Med. 2020;58(7):1029-36.

29. Pizzi R, Gini G, Caiano L, Castelli B, Dotan N, Magni F, et al. Coagulation parameters and venous thromboembolism in patients with and without COVID-19 admitted to the Emergency Department for acute respiratory insufficiency. Thromb Res. 2020;196:209-12.

30. Voicu S, Delrue M, Chousterman BG, Stepanian A, Bonnin P, Malissin I, et al. Imbalance between procoagulant factors and natural coagulation inhibitors contributes to hypercoagulability in the critically ill COVID-19 patient: clinical implications. Eur Rev Med Pharmacol Sci. 2020;24(17):9161-8.

31. Mei H, Hu Y. [Characteristics, causes, diagnosis and treatment of coagulation dysfunction in patients with COVID-19]. Zhonghua Xue Ye Xue Za Zhi. 2020;41(3):185-91.

32. Bao C, Tao X, Cui W, Yi B, Pan T, Young KH, et al. SARS-CoV-2 induced thrombocytopenia as an important biomarker significantly correlated with abnormal coagulation function, increased intravascular blood clot risk and mortality in COVID-19 patients. Exp Hematol Oncol. 2020;9:16.

33. Xiong M, Liang X, Wei YD. Changes in blood coagulation in patients with severe coronavirus disease 2019 (COVID-19): a meta-analysis. Br J Haematol. 2020;189(6):1050-2.
34. Kander T. Coagulation disorder in COVID-19. The Lancet Haematology. 2020;7(9):e630-e2.
35. Song JC, Wang G, Zhang W, Zhang Y, Li WQ, Zhou Z, et al. Chinese expert consensus on diagnosis and treatment of coagulation dysfunction in COVID-19. Mil Med Res. 2020;7(1):19.
36. Chen X, Wang Q, Xu M, Li C. A Retrospective Analysis of the Coagulation Dysfunction in COVID-19 Patients. Clin Appl Thromb Hemost. 2020;26:1076029620964868.
37. Pavoni V, Gianesello L, Pazzi M, Stera C, Meconi T, Frigieri FC. Evaluation of coagulation function by rotation thromboelastometry in critically ill patients with severe COVID-19 pneumonia. J Thromb Thrombolysis. 2020;50(2):281-6.
38. Savioli F, Rocha LL. Coagulation profile in severe COVID-19 patients: what do we know so far? Rev Bras Ter Intensiva. 2020;32(2):197-9.
39. Jose RJ, Manuel A. COVID-19 cytokine storm: the interplay between inflammation and coagulation. Lancet Respir Med. 2020;8(6):e46-e7.
40. Kasinathan G, Sathar J. Haematological manifestations, mechanisms of thrombosis and anti-coagulation in COVID-19 disease: A review. Ann Med Surg (Lond). 2020;56:173-7.

Blood coagulation parameter abnormalities in hospitalized patients with confirmed COVID-19 in Ethiopia

Shambel Araya[1&2], Mintesnot Aragaw Mamo [1&2], Yakob Gebregziabher Tsegay[3&4], Asegdew Atlaw[2], Aschalew Aytenew[2], Abebe Hordofa[2], Abebe Edao Negeso[1], MogesWordofa[1], Tirhas Niguse[1], Mahlet Cheru[1], Zemenu Tamir[1]

[1]Department of Medical Laboratory Sciences, College of Health Sciences, Addis Ababa University, Addis Ababa, Ethiopia

[2]Department of Medical Laboratory, Millennium COVID-19 Treatment and Care Centre, St. Paual Millennium Medical College, Addis Ababa, Ethiopia;

[3]Department of Medical Biotechnology, Institute of Biotechnology, University of Gondar, Gondar, Ethiopia.

[4]Department of Research and development center, College of Health Sciences, Defense University, Addis Ababa, Ethiopia.

Address:

1. Shambel Araya (corresponding author);shambelaraya8@gmail.com
2. Mintsnot Aragaw Mamo:mintsh2015@gmail.com
3. Yakob Gebreegziabher Tsegaye: yakobtsegay17@gmail.com
4. Asegdew Atlaw: asegdew21@gmail.com
5. AschalewAytenew:aschu9033@gmail.com
6. Abebe Hordofa: abuhordofa@gmail.com
7. Abebe EdaoNegeso:abenegesso@gmail.com
8. Moges Wordofa: heranmakmow@gmail.com
9. TirhasNiguse;peace.for.all.060610@gmail.com
10. MahletCheru:yuluyaya54@gmail.com
11. Zemenu Tamir: zemenut266@gmail.com

Abstract

Background: Coagulopathy and thromboembolic events are among the complications of Corona Virus disease 2019 (COVID-19). Thus, abnormal coagulation profiles in COVID-19 patients are taken as important prognostic factors of COVID-19 disease severity. The aim of this study was to analyze coagulation profiles of hospitalized COVID-19 patients in Addis Ababa, Ethiopia. **Methods**: This cross-sectional study was conducted among 455 Covid-19 patients admitted at Millennium COVID-19 treatment center, Addis Ababa, Ethiopia from July 1- October 23, 2020. Prothrombin Time (PT), Activated Partial Thromboplastin Time (APTT) and International normalized ratio (INR) were ~~estimated by~~determined on HUMACLOT DUE PLUS coagulation analyzer (Wiesbaden, Germany). In all tests, $p<0.05$ was defined as statistically significant.

Result: A prolonged prothrombin time was found in~~among~~ 46.8% of study subjects with COVID-19 and a prolonged prothrombin time and elevated INR with 53.3% of study subjects with severe and 51 % of critically COVID patients.~~. Prolonged prothrombin time and high INR were seen among 53.3% severe and 51% critical patients with COVID-19 manifestation.~~ Thrombocytopenia was detected in 22.1% of COVID-19 patients. 50.5% and 51.3% of COVID-19 patients ~~aged~~ older than 55 years had thrombocytopenia and prolonged APTT respectively.

Conclusion:

In this study, prolonged prothrombin time and high INR were detected in around 50% of severe ~~found among severe~~ and critical COVID-19 patients. Thrombocytopenia and prolonged APTT were dominant in COVID-19 patients older than 55 years. Thus, we recommend emphasis to be given for monitoring of platelet count, PT, APTT and INR in hospitalized COVID-19 patients ~~management~~.

Key words: COVID-19, Prothrombin time, activated partial thromboplastin time, international normalized ratio, Platelet, Addis Ababa, Ethiopia.

Introduction

Coronavirus disease 2019 (COVID-19) is caused by a novel beta corona virus called severe acute respiratory syndrome coronavirus 2 (SARS-CoV-2)(1). COVID-19 has become a pandemic that has heavily affected the global population. As of November 8, 2020, there have been 49,578,590 confirmed cases of COVID-19 and 1,245,717 deaths, reported to World Health organization (WHO). Similarly, there have been 99,204 confirmed cases of COVID-19 with 1,518 deaths in Ethiopia(2).

The severity of COVID-19 varies considerably from asymptomatic to life threatening, lung injury being the main clinical manifestation. Most of the patients have a favorable prognosis, but some rapidly progress to severe and critical cases with respiratory distress syndrome, coagulation dysfunction and multiple organ failures (3, 4). Although the pathophysiology and the underlining mechanisms of clinical manifestations remain unclear, thrombo-inflammation and cytokine storm are clearly established components in Severe Acute Respiratory Distress Syndrome (SARS) of COVID-19(5-8).

Coagulopathy and abnormal coagulation profiles were indicated among the most significant markers of poor prognosis in COVID-19 patients (9-11). A retrospective cohort study conducted in Spain Madrid demonstrated that~~showed~~ COVID-19 non-survivors had significantly lower prothrombin activity, abnormal coagulation parameters like prolonged PT, APTT, higher D-dimer and higher fibrinogen levels compared to survivors indicating coagulation parameters could be an efficient measure for ~~improving the clinical management and~~ predicting the prognosis of patients with SARS COV-2(7) and guiding management. Similarly, Long et al has reported that occurrence of coagulation dysfunction is more likely in severe and critically ill patients. The study also showed that D-dimer and prothrombin time could be considered as main

indicators in predicting the mortality of COVID-19 patients(3). Different studies also support that COVID-19 patients are at high risk of developing disseminated intravascular coagulation (12, 13).

Several studies have also demonstrated the increased occurrence of intravascular disseminated coagulopathy (DIC) in patients with COVID-19 (12,13). The result of blood coagulation parameters in COVID-19 can also guide management decisions and improve outcomes (12,14).

It is also indicated that comparison of reports from various populations related to the clinical course, outcome of COVID-19 and blood coagulation profile in these patients are necessary to help the management and treatment of the disease (12, 14). Moreover, this routine coagulation parameter tests results could potentially be utilized in symptomatic patients in resource limited settings with inadequate access to COVID-19 RT-PCR , as in Ethiopia, to raise suspension of this infection. could be used as potential indicators for COVID-19 in individuals having typical clinical manifestations that would be inputs for prompt patient management especially in resource limited settings where the high-tech gold standard RT-PCR is not widely available, like Ethiopia. However, data on coagulation profiles among Ethiopian COVID-19 patients is not readily availablescarce. Thus, the aim of this study was to determinefind out the coagulation profile of COVID-19 patients admitted at Millennium COVID-19 treatment center, Addis Ababa, Ethiopia.

Methods

Ethical consideration: The study was approved by Addis Ababa University College of Health Sciences, department of Medical Laboratory Sciences research ethics review committee (DRERC/538/20/MLS) and it was in accordance with the principles of the Helsinki II

declaration. Laboratory test results were communicated to the responsible clinicians working at the treatment center. Written informed consent was obtained from the study participants. All the information obtained from the study participants were kept confidential.

Study population

In this study, we have included 455 consecutive patients with confirmed SARS-CoV-2 infection ~~and~~ admitted to Millennium COVID-19 treatment center, Addis Ababa, Ethiopia from July 1-October 23, 2020. The treatment center is the first referral center for~~of~~ COVID-19 patients in Ethiopia, since May 02, 2020. None of the study participants were ~~receving~~receiving~~was taking~~ anticoagulant medications before blood drawing. Diagnosis of SARS-CoV-2 infection was made with ~~according to~~ real time PCR.

Sample collection and coagulation profile analysis

2.6. Laboratory Analysis

~~Eight milliliters of v~~Venous blood were collected by professional nurses working in the treatment center: 5 mL~~five milliliters~~ in EDTA ~~for platelet count, three milliliters~~and 3 mL in 3.2% sodium citrated anti-coagulated tube for analysis of coagulation parameters. The samples for coagulation tests were collected at hospital admission. The prothrombin time (PT), activated partial prothrombin time (APTT), and international normalized ratio (INR) were analyzed using HUMACLOT DUE PLUS® coagulation analyzer (Wiesbaden ®, Germany). Platelet count was performed using UniCel® DxH 800 Coulter®Cellular Analysis System (Beckman Coulter ®, Inc. 4300 N. Harbor Blvd. Fullerton, CA 92835). The coagulation parameters were compared with the manufacturer cut off normal range of PT = 11.7- 15 seconds,

APTT = 23.8- 37.9 seconds, INR = 1.0- 1.2 and PLT= 159-386/µ.l. The coagulation parameters above the cut off range were considered as a prolonged time and thrombocytopenia in the case of lower than cut off value for platelet. All laboratory tests and its interpretation were done following the manufacturers' recommendation and standard operating procedures.

Statistical Analysis

Statistical Package for the Social Sciences (SPSS) software version 25.0 (SPSS® Inc., Chicago, IL, USA) was used for statistical analysis. Chi-square test was used to determine association among categorical variables. The quantitative data were expressed as Mean ± SD. P value < 0.05 was considered as statistically significant.

Results

Socio-demographic and Clinical characteristics of Study participants

In this study, 455 patients diagnosed with COVID-19 were included. Among the study participants, 289 (63.5%) were males. The study participants were between the age of 4 and 90 years with a mean of 49.9 ±18.3 years. From the total 455 case, there were 297 mild cases, 90 severe cases, and 68 critical cases based on disease severity of COVID-19 (Table 1).

Table 1: Socio-demographic characteristics of study participants ~~Addis Ababa, Ethiopia, 2020~~

Variables		Frequency	Percent
Gender	Male	289	63.5%
	Female	166	36.5%
Age group	0-18 years	15	3.2%

	18-35 years	101	22.1%
	36-55 years	158	34.7%
	>55 years	181	39.7%
Disease severity	Moderate	297	65.2%
	Severe	90	19.8%
	Critical	68	15%

The median time from the disease onset to admission was 4 days (2-8 days). Severe and critical groups showed differences in sex ratio and age distribution. In ~~severe~~severe (36.6%) and critical groups (48.5%), ~~majority~~ were elderly males of the age of >55 years old. ~~males and elderl~~y (Table 2).

Table 2: Socio-demographic characteristics and disease severity of COVID-19 patients ~~admitted to Millennium COVID-19 treatment center Addis Ababa, Ethiopia~~

Variables		Disease Severity			P-value
		Moderate, n (%)	Severe, n (%)	Critical, n (%)	
Age(year)	0-18, n= 15	10(66.7)	4(26.7)	1(6.7)	0.283
	18-35, n=101	65(64.35)	22(21.78)	14(13.8)	
	36-55, n=158	107(67.7)	31(19.6)	20(12.65)	
	>55, n=181	115(63.5)	33(18.2)	33(8.2)	
Sex, N=455	Male, n=289	187(64.7)	56(19.3)	46(15.9)	0.045
	Female, n=166	110(66.2)	34(20.4)	22(13.2)	

Magnitude of coagulation abnormalities

In this study, 209 COVID-19 patients (46%) showed prolonged PT and higher INR values. Among those patients with prolonged PT, 51.3% were above 55 years of age. Prolonged PT values were demonstrated more ~~was found among~~frequently among males (49.8%) than females (41%) and this difference was statistically different ~~and it has a significant association with gender~~ (P = 0.045). Similarly, 51.4% and 53.3% of ICU (critical) and severe patients had prolonged PT values. Notably, prolonged APTT values were found among 43.1% of individuals, and from these 47%, 45% and 41% were among ICU (critical), severe and moderate patients, respectively. 57.2% of female patients had prolonged APTT; and 51.3% of patients aged older than 55 years had a prolonged APTT.

Thrombocytopenia was detected in 22.1% (101 out of 455) study subjects~~22.1% individuals~~. 50.5% (50 out of 99) patients aged older than 55 years had thrombocytopenia and the occurrence~~. Thrombocytopenia~~ was higher among male (23.8%) than female (18%) ICU patients (Table 3).

Coagulation Parameters		***Variables***								
		Age				***Sex***		***Disease Severity***		
		0-18 n(%)	***19-35 n(%)***	***36-55 n(%)***	***>55 n(%)***	***Male n(%)***	***Female n(%)***	***Moderate n(%)***	***Severe n(%)***	***Critical n(%)***
PT	High n=213	9(4.2)	50(23.47)	61(28.6	93(43.6)	144(67.6)	69(32.4)	130(61)	48(22.5)	35(16.4)
	Normal n=220	6(2.7)	45(20.45)	89(40.4)	80(36.3)	131(59.5)	89(40.4)	149(67.7)	40(18.1)	31(14.1)
	Low=22	0	6(27.2)	8(36.3)	8(36.3)	14(63.6)	8(36.3)	18(81)	2(9)	2(9)
APTT	High=196	6(3)	46(23.4)	68(34.7)	76(38.77)	101(51.5)	95(48.5)	115(58.67)	41(21)	42(21.4)
	Normal	6(3.1)	38(19.7)	70(36.2)	79(41)	136(70.4)	57(29.5)	137(71)	36(18.6)	21(10.8)

	n=193									
	Low n=66	3(4.5)	17(25.7)	20(30.3)	26(39.3)	52(78.7)	14(21)	45(68)	13(19.7)	5(7.5)
PLT	High n=65	4(6.1)	11(17)	24(37)	26(40)	43(66)	22(33.8)	39(60)	8(12.3)	8(12.3)
	Normal n=289	8(3)	70(24.2)	105(36.3)	105(36.3)	175(60.8)	114(39)	214(74)	44(15)	31(10.6)
	Low n= 101	3(2.9)	20(20)	28(27.7)	50(49.5)	69(69.70	30(30.3)	33(32)	38(37.6)	30(29.7)
INR	High n=210	9(4.2)	50(24.7)	60(28.5)	91(43.3)	141(67)	69(32.8)	127(60.4)	50(23.8)	33(15.7)
	Normal =224	5(2.2)	44(19.6)	93(41.5)	82(36.6)	115(51)	75(33.4)	113(50.4)	45(20)	32(14.2)
	Low n=21	1(4.7)	7(33.3)	5(23.8)	8(38)	14(66.6)	7(33.3)	15(71)	3(14.5)	3(14.5)

Table 3: Result of c~~C~~oagulation parameters in patients with severe COVID-19 according to different variables

PLT=platelet; PT= prothrombin time; APTT=activated partial thromboplastin time; INR=international normalized ratio.

Discussion

The COVID-19 pandemic ~~has brought~~had a major impact on health care globally. COVID-19 has already caused >1.2 million deaths worldwide and more than 1400 in Ethiopia as of October 30,2020 according to WHO report(15). Coagulation abnormalities are ~~indicated as~~ frequent ~~findings~~ in COVID-19 patients and are associated with poor prognosis and reduced survival(7). ~~Similarly, it is also indicated that coagulopathy which is resulted due to~~ The dysregulation of coagulation and associated with hypercoagulability in patients with COVID manifest as ~~as evidenced by~~ venous and arterial thrombosis and multiorgan dysfunction(16); which are poor prognostic markers resulting in increased mortality and hospitalization and ICU admission ~~is one of the most significant prognostic factors in patients with COVID-19 and is associated with increased hospitalization, admission to critical care, and mortality~~(14, 17-19). Previous studies

indicated that coagulopathy in patients hospitalized with COVID-19 is characterized by increase in coagulation parameters such as PT, APTT and INR ~~levels~~ (20, 21).

Patients with serious infection are more likely to have COVID-19 associated coagulopathy than patients with a mild infection (21,22). In our study, prolonged PT, APTT an INR was more frequent~~found~~ among severe and critical COVID-19 patients ~~than moderate ones~~. Similarly, studies also reported that thrombotic complications are common among COVID-19 patients admitted to intensive care unit (ICU) (9.5%-47%)(22-24).

Treatment of the underlying condition is suggested to be paramount in coagulopathies. It is shown that bleeding is not common clinical manifestation in COVID-19 infections despite abnormal coagulation parameters (23,24). ~~Along these, it is suggested that~~and supportive care including blood product transfusion should be individualized in COVID -19 patients(25, 26). Laboratory findings alone should not be taken as basis for instituting blood transfusion therapy, rather it should be reserved for those who are bleeding, requires an invasive procedure, or who are otherwise at high risk for bleeding complications (26, 27).

Published studies~~Evidences~~ indicate that COVID-19 is associated with a hyper-coagulable state. Venous thromboembolism (VTE) and arterial thrombosis ranging from 15% to 30% were found in critically ill patients with COVID-19 and about 7% in those admitted to medical wards (28-30). ~~Clotting is reported from~~Abnormal thrombosis of different medical devices~~ used~~, deep vein thrombosis and multiple thrombi in the vessels of the lungs, kidneys and other organs at~~from~~ autopsy of patients who died of Covid-19 have been reported serving as the impetus behind guidelines -(9, 29)~~.~~ which support the use of ~~These indicate clinicians to use~~ therapeutic doses of heparin or low-molecular-weight heparin instead of prophylactic doses in critically ill COVID-19 patients (12, 26, 31). In the current study, thrombocytopenia was observed among males

(23.8%) than females (19.8%) and older people (27.6%). Severe (42.68%) and critical (42%) patients also had thrombocytopenia and this was in line with studies conducted in different countries (20, 22, 32, 33). Thrombocytopenia, defined as platelet count less than 100×10^{9} cells/L were independently associated with COVID-19 severity(34). Studies ~~across~~ suggest~~ed~~ that routine coagulation test results~~s~~ ~~can~~ ~~be considered~~ are markers of disease severity and assist in management decision.~~as a significant marker to help clinicians assess prognosis and severity of patients with COVID-19~~. In critically ill patients, thrombocytopenia correlates with multi-organ failure and death, and a decline in platelet count, even in the absence of overt thrombocytopenia, portends a worse outcome (9, 12, 13). In patients who are not bleeding, there is no evidence that correction of laboratory parameters with blood products improves outcomes. Replacement might worsen disseminated thrombosis and further deplete scarce blood products (28, 35).

Many studies reported that coagulopathy associated with COVID-19 is characterized by thrombocytopenia, prolongation of the prothrombin time, high levels of D-dimer, and elevated levels of fibrinogen, factor VIII, and von Willebrand factor (3, 11, 16). The degree of coagulation abnormalities in critically ill non-COVID patients correlates with disease severity and predict the risk of thrombosis, the need for ventilator support, and mortality. Published studies support that COVID-19-associated coagulopathy is ~~however unique~~characterized by ~~with a much~~ a decreased platelet ~~count~~ count (9, 36-38). Patients with critical COVID-19 infection and a cytokine storm have an extreme hyper-coagulable state. Even though the reason for this life-threatening condition is not known, this might be due to an uncontrolled hyper-inflammatory response without previous immunity (39, 40).

Conclusion: In this study, prolonged prothrombin time and high INR were found among severe and critical COVID-19 patients. Thrombocytopenia and prolonged APTT were dominant in COVID-19 patients older than 55 years. Thus, we recommend emphasis to be given for monitoring of platelet count, PT, APTT and INR in hospitalized COVID-19 patients management.

~~**Ethical Clearance**: Ethical clearance was obtained from Addis Ababa University, College of Health Science, Department of Medical Laboratory Sciences, Research ethics review committee and it was in accordance with the principles of the Helsinki II declaration.~~

Consent for publication: Not applicable

Availability of data and material

All the available data were included in the manuscript.

Funding: None.

Conflict of interest: The authors declare that they have no conflict of interest.

References

1. Sohrabi C, Alsafi Z, O'Neill N, Khan M, Kerwan A, Al-Jabir A, et al. World Health Organization declares global emergency: A review of the 2019 novel coronavirus (COVID-19). Int J Surg. 2020;76:71-6.
2. World Health Organization. Who coronavirus disease (covid-19) dashboard 2020,November 8 [Available from: https://covid19.who.int/info.
3. Long H, Nie L, Xiang X, Li H, Zhang X, Fu X, et al. D-Dimer and Prothrombin Time Are the Significant Indicators of Severe COVID-19 and Poor Prognosis. BioMed research international. 2020;2020:6159720.
4. Guan WJ, Ni ZY, Hu Y, Liang WH, Ou CQ, He JX, et al. Clinical Characteristics of Coronavirus Disease 2019 in China. The New England journal of medicine. 2020;382(18):1708-20.
5. Aggarwal M, Dass J, Mahapatra M. Hemostatic Abnormalities in COVID-19: An Update. Indian J Hematol Blood Transfus. 2020:1-11.

6. Coccheri S. COVID-19: The crucial role of blood coagulation and fibrinolysis. Intern Emerg Med. 2020;15(8):1369-73.
7. Quintana-Díaz M, Andrés-Esteban EM, Ramírez-Cervantes KL, Olivan-Blázquez B, Juárez-Vela R, Gea-Caballero V. Coagulation Parameters: An Efficient Measure for Predicting the Prognosis and Clinical Management of Patients with COVID-19. Journal of Clinical Medicine. 2020;9(11).
8. Parasher A. COVID-19: Current understanding of its pathophysiology, clinical presentation and treatment. Postgrad Med J. 2020.
9. Al-Samkari H, Karp Leaf RS, Dzik WH, Carlson JCT, Fogerty AE, Waheed A, et al. COVID-19 and coagulation: bleeding and thrombotic manifestations of SARS-CoV-2 infection. Blood. 2020;136(4):489-500.
10. Annunziata A, Imitazione P, Polistina GE, Lanza M, Coppola A, Fiorentino G. Pulmonary Embolism in Covid-19: Coagulation Parameters, Close Monitoring to Prevent? Turk Thorac J. 2020;21(4):287-8.
11. Savioli F, Rocha LL. Coagulation profile in severe COVID-19 patients: what do we know so far? Revista Brasileira de Terapia Intensiva. 2020;32(2).
12. Buioni D, Nardi P, Ruvolo G. Thrombocytopenia and coagulation disorders due to COVID 19 infection with concomitant cardiovascular diseases requiring anti-platelet and anticoagulant therapy, which strategy? Clin Chim Acta. 2020;508:109.
13. Levi M. COVID-19 coagulopathy vs disseminated intravascular coagulation. Blood Adv. 2020;4(12):2850.
14. Quintana-Diaz M, Andres-Esteban EM, Ramirez-Cervantes KL, Olivan-Blazquez B, Juarez-Vela R, Gea-Caballero V. Coagulation Parameters: An Efficient Measure for Predicting the Prognosis and Clinical Management of Patients with COVID-19. J Clin Med. 2020;9(11).
15. WHO. COVID19. 2020. Contract No.:

.
16. Chan NC, Weitz JI. COVID-19 coagulopathy, thrombosis, and bleeding. Blood. 2020;41:100648.
17. Rauch A, Labreuche J, Lassalle F, Goutay J, Caplan M, Charbonnier L, et al. Coagulation biomarkers are independent predictors of increased oxygen requirements in COVID-19. J Thromb Haemost. 2020.
18. Zhang Y, He L, Chen H, Lu S, Xiong Y, Liu J, et al. Manifestations of blood coagulation and its relation to clinical outcomes in severe COVID-19 patients: Retrospective analysis. Int J Lab Hematol. 2020.
19. Zhang A, Leng Y, Zhang Y, Wu K, Ji Y, Lei S, et al. Meta-analysis of coagulation parameters associated with disease severity and poor prognosis of COVID-19. International journal of infectious diseases : IJID : official publication of the International Society for Infectious Diseases. 2020;100:441-8.
20. Luo HC, You CY, Lu SW, Fu YQ. Characteristics of coagulation alteration in patients with COVID-19. Ann Hematol. 2020.
21. Levi M, Thachil J, Iba T, Levy JH. Coagulation abnormalities and thrombosis in patients with COVID-19. Lancet Haematol. 2020;7(6):e438-e40.
22. Zou Y, Guo H, Zhang Y, Zhang Z, Liu Y, Wang J, et al. Analysis of coagulation parameters in patients with COVID-19 in Shanghai, China. Biosci Trends. 2020;14(4):285-9.

23. Sayad B, Rahimi Z. Blood coagulation parameters in patients with severe COVID-19 from Kermanshah Province, Islamic Republic of Iran. East Mediterr Health J. 2020;26(9):999-1004.
24. Adam EH, Zacharowski K, Miesbach W. A comprehensive assessment of the coagulation profile in critically ill COVID-19 patients. Thromb Res. 2020;194:42-4.
25. Paar V, Wernly B, Zhou Z, Motloch LJ, Hoppe UC, Egle A, et al. Anti-coagulation for COVID-19 treatment: both anti-thrombotic and anti-inflammatory? J Thromb Thrombolysis. 2020.
26. Belen-Apak FB, Sarialioglu F. Pulmonary intravascular coagulation in COVID-19: possible pathogenesis and recommendations on anticoagulant/thrombolytic therapy. J Thromb Thrombolysis. 2020;50(2):278-80.
27. Lee AY, Connors JM, Kreuziger LB, Murphy M, Gernsheimer T, Lin Y, et al. COVID-19 and Coagulopathy 2020, December 1 [Available from: https://www.hematology.org/COVID-19/COVID-19-and-coagulopathy.
28. Harenberg J, Favaloro E. COVID-19: progression of disease and intravascular coagulation - present status and future perspectives. Clin Chem Lab Med. 2020;58(7):1029-36.
29. Pizzi R, Gini G, Caiano L, Castelli B, Dotan N, Magni F, et al. Coagulation parameters and venous thromboembolism in patients with and without COVID-19 admitted to the Emergency Department for acute respiratory insufficiency. Thromb Res. 2020;196:209-12.
30. Voicu S, Delrue M, Chousterman BG, Stepanian A, Bonnin P, Malissin I, et al. Imbalance between procoagulant factors and natural coagulation inhibitors contributes to hypercoagulability in the critically ill COVID-19 patient: clinical implications. Eur Rev Med Pharmacol Sci. 2020;24(17):9161-8.
31. Mei H, Hu Y. [Characteristics, causes, diagnosis and treatment of coagulation dysfunction in patients with COVID-19]. Zhonghua Xue Ye Xue Za Zhi. 2020;41(3):185-91.
32. Bao C, Tao X, Cui W, Yi B, Pan T, Young KH, et al. SARS-CoV-2 induced thrombocytopenia as an important biomarker significantly correlated with abnormal coagulation function, increased intravascular blood clot risk and mortality in COVID-19 patients. Exp Hematol Oncol. 2020;9:16.
33. Xiong M, Liang X, Wei YD. Changes in blood coagulation in patients with severe coronavirus disease 2019 (COVID-19): a meta-analysis. Br J Haematol. 2020;189(6):1050-2.
34. Kander T. Coagulation disorder in COVID-19. The Lancet Haematology. 2020;7(9):e630-e2.
35. Song JC, Wang G, Zhang W, Zhang Y, Li WQ, Zhou Z, et al. Chinese expert consensus on diagnosis and treatment of coagulation dysfunction in COVID-19. Mil Med Res. 2020;7(1):19.
36. Chen X, Wang Q, Xu M, Li C. A Retrospective Analysis of the Coagulation Dysfunction in COVID-19 Patients. Clin Appl Thromb Hemost. 2020;26:1076029620964868.
37. Pavoni V, Gianesello L, Pazzi M, Stera C, Meconi T, Frigieri FC. Evaluation of coagulation function by rotation thromboelastometry in critically ill patients with severe COVID-19 pneumonia. J Thromb Thrombolysis. 2020;50(2):281-6.
38. Savioli F, Rocha LL. Coagulation profile in severe COVID-19 patients: what do we know so far? Rev Bras Ter Intensiva. 2020;32(2):197-9.
39. Jose RJ, Manuel A. COVID-19 cytokine storm: the interplay between inflammation and coagulation. Lancet Respir Med. 2020;8(6):e46-e7.

40. Kasinathan G, Sathar J. Haematological manifestations, mechanisms of thrombosis and anti-coagulation in COVID-19 disease: A review. Ann Med Surg (Lond). 2020;56:173-7.
