# Supplementary material for: Blood coagulation parameter abnormalities in hospitalized patients with confirmed COVID-19 in Ethiopia
Source: PLoS One. 2021 Jun 21;16(6):e0252939. doi: 10.1371/journal.pone.0252939 (PMC8216564; doi:10.1371/journal.pone.0252939)
Supplement: Attachment — Submitted filename: PONE-D-20-35898_R3_reviewer SL.pdf [file pone.0252939.s004.pdf]

PLOS ONE

Blood coagulation parameter abnormalities in hospitalized patients with confirmed COVID-19 in Ethiopia

--Manuscript Draft--

**Manuscript Number:**	PONE-D-20-35898R3
**Article Type:**	Research Article
**Full Title:**	Blood coagulation parameter abnormalities in hospitalized patients with confirmed COVID-19 in Ethiopia
**Short Title:**	Coagulation profile of COVID-19 patients
**Corresponding Author:**	Shambel Araya<br>Addis Ababa University<br>Addis Ababa, ETHIOPIA
**Keywords:**	COVID-19; Prothrombin time; activated partial thromboplastin time; international normalized ratio; platelet; Addis Ababa; Ethiopia
**Abstract:**	Background : Coagulopathy and thromboembolic events are among the complications of Corona Virus disease 2019 (COVID-19). Thus, abnormal coagulation profiles in COVID-19 patients are taken as important prognostic factors of COVID-19 disease severity. The aim of this study was to analyze coagulation profiles of hospitalized COVID-19 patients in Addis Ababa, Ethiopia. Methods : This cross-sectional study was conducted among 455 Covid-19 patients admitted at Millennium COVID-19 treatment center, Addis Ababa, Ethiopia from July 1- October 23, 2020. Prothrombin Time (PT), Activated Partial Thromboplastin Time (APTT) and International normalized ratio (INR) were determined on HUMACLOT DUE PLUS coagulation analyzer (Wiesbaden, Germany) . In all tests, p<0.05 was defined as statistically significant.<br>Result : A prolonged prothrombin time was found in 46.8% of study subjects with COVID-19 and a prolonged prothrombin time and elevated INR with 53.3% of study subjects with severe and 51 % of critically COVID patients. Thrombocytopenia was detected in 22.1% of COVID-19 patients. 50.5% and 51.3% of COVID-19 patients older than 55 years had thrombocytopenia and prolonged APTT respectively.<br>Conclusion :<br>In this study, prolonged prothrombin time and high INR were detected in around 50% of severe and critical COVID-19 patients. Thrombocytopenia and prolonged APTT were dominant in COVID-19 patients older than 55 years. Thus, we recommend emphasis to be given for monitoring of platelet count, PT, APTT and INR in hospitalized COVID-19 patients.
**Order of Authors:**	Shambel Araya, MSc
	Mintesnot Aragaw Mamo
	Yakob Gebregziabher Tsegay
	Aschalew Aytenew
	Abebe Hordofa
	AbebeEdao Negeso
	Zemenu Tamir
	Tirhas Niguse
	Mahlet Cheru
	Asegdew Atlaw
	Moges Wordofa
**Response to Reviewers:**	General Comments<br>The reviewer felt that the manuscript was substantially improved but there are still a number of grammatical and typographical errors that should be corrected. Again, it is recommended that the manuscript is reviewed by a native English language speaker.

Powered by Editorial Manager® and ProduXion Manager® from Aries Systems Corporation

	Dear reviewers and editors<br>We have carefully consider your comments and we have incorporated all your comments and recommendations<br>The manuscript have also reviewed by a native English language speaker<br>Specific comments<br>Comment 1: Abstract, background section:<br>“Thus”, “taken as” and “COVID-19” was removed as per your suggestion in line 28 & 29<br>Comment 2: Abstract, method section:<br>Insert trade mark: inserted<br>Remove tests and replace it with statistical analysis results: accepted and replaced<br>Comment 3: Result section: “with” is replaced with ‘in’ in line<br>Comment 4: abstract, conclusion section.<br>Replace “around” with “more than”<br>Comment5: Introduction section:<br>Dear editor and reviewers, all comments and suggestions given in introduction section were accepted and amended accordingly<br>Remove “heavily” in line 52<br>Replace 49,578, 490 with “more than 1.4 million” in line 53<br>Insert with lung injury in line 56<br>Remove and critical cases with<br>Comment 6: method section:<br>Dear editor and reviewers, all comments and suggestions given in method section were accepted and amended accordingly<br><br>Insert “personal identifying” in line 87: accepted and inserted<br>Delete “time”, “its” in line 107 and 108<br>Replace Platelet with platelets in line 108<br>Comment 6: Result section:<br>Dear editor and reviewers, all comments and suggestions given in result section were accepted and amended accordingly<br>Delete Y in line 126<br>Delete N=455 in table 1<br>Replace “higher” with “elevated”<br>Move heading of table 3 to the top<br>Comment 6: Discussion section:<br>Dear editor and reviewers, all comments and suggestions given in discussion section were accepted and amended accordingly<br>Replace “and” with “is” in line 153<br>Replace in “patients with COVID” with “and” in line 151<br>Delete “resulting in increased mortality and hospitalization and ICU admission” in line 153<br>Change manifest in to manifests and increase in to increases<br>Delete “(9.5%-47%)” in line 160<br>Insert “More frequently” in line 176 and 177<br>Rephrase the sentences from line 188-193<br>Before: The degree of coagulation abnormalities in critically ill non-COVID patients correlates with disease severity and predict the risk of thrombosis, the need for ventilator support, and mortality. Published studies support that COVID-19-associated coagulopathy is characterized by a decreased platelet count (9, 36-38). Patients with critical COVID-19 infection and a cytokine storm have an extreme hyper-coagulable state.<br>Modified: Published studies indicate that COVID-19-associated coagulopathy is characterized by a decreased platelet count (9, 36-38)and a cytokine storm with an extreme hyper-coagulable state.<br>Comment 6: Discussion section:<br>Replace “APTT” with “clotting time assays”<br>Response: Dear editors and reviewers all of the above listed comments, recommendations and suggestions are accepted, replaced and corrected and we thank you very much for your valuable comments & time.<br>Looking forward to hearing from you. Thank you again for your consideration!<br>Sincerely,<br>Shambel Araya (BSc, MSc, PhD fellow)
**Additional Information:**	

Powered by Editorial Manager® and ProduXion Manager® from Aries Systems Corporation

Question	Response
**Financial Disclosure**<br><br>Enter a financial disclosure statement that describes the sources of funding for the work included in this submission. Review the submission guidelines for detailed requirements. View published research articles from *PLOS ONE* for specific examples.<br><br>This statement is required for submission and **will appear in the published article** if the submission is accepted. Please make sure it is accurate.<br><br>**Unfunded studies**<br>Enter: *The author(s) received no specific funding for this work.*<br><br>**Funded studies**<br>Enter a statement with the following details:<br>• Initials of the authors who received each award<br>• Grant numbers awarded to each author<br>• The full name of each funder<br>• URL of each funder website<br>• Did the sponsors or funders play any role in the study design, data collection and analysis, decision to publish, or preparation of the manuscript?<br>• **NO** - Include this sentence at the end of your statement: *The funders had no role in study design, data collection and analysis, decision to publish, or preparation of the manuscript.*<br>• **YES** - Specify the role(s) played.<br><br>* typeset	The author(s) received no specific funding for this work.
**Competing Interests**<br><br>Use the instructions below to enter a competing interest statement for this submission. On behalf of all authors, disclose any competing interests that could be perceived to bias this work—acknowledging all financial support and any other relevant financial or non-financial competing interests.<br><br>This statement **will appear in the published article** if the submission is	The authors have declared that no competing interests exist

accepted. Please make sure it is accurate. View published research articles from *PLOS ONE* for specific examples.<br><br>**NO authors have competing interests**<br><br>Enter: *The authors have declared that no competing interests exist.*<br><br>**Authors with competing interests**<br><br>Enter competing interest details beginning with this statement:<br><br>*I have read the journal's policy and the authors of this manuscript have the following competing interests: [insert competing interests here]*<br><br>* typeset	
**Ethics Statement**<br><br>Enter an ethics statement for this submission. This statement is required if the study involved:<br><br>• Human participants<br>• Human specimens or tissue<br>• Vertebrate animals or cephalopods<br>• Vertebrate embryos or tissues<br>• Field research<br><br>Write "N/A" if the submission does not require an ethics statement.<br><br>General guidance is provided below. Consult the submission guidelines for detailed instructions. **Make sure that all information entered here is included in the Methods section of the manuscript.**	Addis Ababa University College of Health Science Department of Medical Laboratory Science research ethics review committee (DRERC) protocol number: DRERC/538/20/MLS

Powered by Editorial Manager® and ProduXion Manager® from Aries Systems Corporation

**Format for specific study types**<br><br>**Human Subject Research (involving human participants and/or tissue)**<br>• Give the name of the institutional review board or ethics committee that approved the study<br>• Include the approval number and/or a statement indicating approval of this research<br>• Indicate the form of consent obtained (written/oral) or the reason that consent was not obtained (e.g. the data were analyzed anonymously)<br><br>**Animal Research (involving vertebrate animals, embryos or tissues)**<br>• Provide the name of the Institutional Animal Care and Use Committee (IACUC) or other relevant ethics board that reviewed the study protocol, and indicate whether they approved this research or granted a formal waiver of ethical approval<br>• Include an approval number if one was obtained<br>• If the study involved *non-human primates*, add *additional details* about animal welfare and steps taken to ameliorate suffering<br>• If anesthesia, euthanasia, or any kind of animal sacrifice is part of the study, include briefly which substances and/or methods were applied<br><br>**Field Research**<br><br>Include the following details if this study involves the collection of plant, animal, or other materials from a natural setting:<br>• Field permit number<br>• Name of the institution or relevant body that granted permission	
**Data Availability**<br><br>Authors are required to make all data underlying the findings described fully available, without restriction, and from the time of publication. PLOS allows rare exceptions to address legal and ethical concerns. See the PLOS Data Policy and FAQ for detailed information.	Yes - all data are fully available without restriction

A Data Availability Statement describing where the data can be found is required at submission. Your answers to this question constitute the Data Availability Statement and **will be published in the article**, if accepted.<br><br>**Important:** Stating 'data available on request from the author' is not sufficient. If your data are only available upon request, select 'No' for the first question and explain your exceptional situation in the text box.<br><br>Do the authors confirm that all data underlying the findings described in their manuscript are fully available without restriction?	
**Describe where the data may be found in full sentences. If you are copying our sample text, replace any instances of XXX with the appropriate details.**<br><br>• If the data are **held or will be held in a public repository**, include URLs, accession numbers or DOIs. If this information will only be available after acceptance, indicate this by ticking the box below. For example: *All XXX files are available from the XXX database (accession number(s) XXX, XXX.*).<br>• If the data are all contained **within the manuscript and/or Supporting Information files**, enter the following: *All relevant data are within the manuscript and its Supporting Information files.*<br>• If neither of these applies but you are able to provide **details of access elsewhere**, with or without limitations, please do so. For example:<br><br>*Data cannot be shared publicly because of [XXX]. Data are available from the XXX Institutional Data Access / Ethics Committee (contact via XXX) for researchers who meet the criteria for access to confidential data.*<br><br>*The data underlying the results presented in the study are available from (include the name of the third party*	All relevant data are within the manuscript

Powered by Editorial Manager® and ProduXion Manager® from Aries Systems Corporation

and contact information or URL).<br>• This text is appropriate if the data are owned by a third party and authors do not have permission to share the data.<br><br>* typeset	
Additional data availability information:	

Manuscript Click here to access/download;Manuscript;Manuscript.doc

Blood coagulation parameter abnormalities in hospitalized patients with confirmed COVID-19 in Ethiopia

Shambel Araya[1&2*], Mintesnot Aragaw Mamo [1&2], Yakob Gebregziabher Tsegay[3&4], Asegdew Atlaw[2], Aschalew Aytenew[2], Abebe Hordofa[2], Abebe Edao Negeso[1], MogesWordofa[1], Tirhas Niguse[1], Mahlet Cheru[1],Zemenu Tamir[1]

[1]Department of Medical Laboratory Sciences, College of Health Sciences, Addis Ababa University, Addis Ababa, Ethiopia

[2]Department of Medical Laboratory, Millennium COVID-19 Treatment and Care Centre, St. Paual Millennium Medical College, Addis Ababa, Ethiopia;

[3]Department of Medical Biotechnology, Institute of Biotechnology, University of Gondar, Gondar, Ethiopia.

[4]Department of Research and development center, College of Health Sciences, Defense University, Addis Ababa, Ethiopia.

Address:

1. Shambel Araya (corresponding author);shambelaraya8@gmail.com
2. Mintsnot Aragaw Mamo:mintsh2015@gmail.com
3. Yakob Gebreegziabher Tsegaye: yakobtsegay17@gmail.com
4. Asegdew Atlaw: asegdew21@gmail.com
5. AschalewAytenew:aschu9033@gmail.com
6. Abebe Hordofa: abuhordofa@gmail.com
7. Abebe EdaoNegeso:abenegesso@gmail.com
8. Moges Wordofa: heranmakmow@gmail.com
9. TirhasNiguse;peace.for.all.060610@gmail.com
10. MahletCheru:yuluyaya54@gmail.com
11. Zemenu Tamir: zemenut266@gmail.com

Abstract

Background: Coagulopathy and thromboembolic events are among the complications of Corona Virus disease 2019 (COVID-19). Abnormal coagulation parameters in COVID-19 patients are important prognostic factors of disease severity. The aim of this study was to analyze coagulation profiles of hospitalized COVID-19 patients in Addis Ababa, Ethiopia. **Methods**: This prospective cross-sectional study was conducted among 455 Covid-19 patients admitted at Millennium COVID-19 care and treatment center, Addis Ababa, Ethiopia from July 1- October 23, 2020. Prothrombin Time (PT), Activated Partial Thromboplastin Time (APTT) and International normalized ratio (INR) were determined on HUMACLOT DUE PLUS® coagulation analyzer (Wiesbaden, Germany). In all statistical analysis of results, $p<0.05$ was defined as statistically significant.

Result: A prolonged prothrombin time was found in 46.8% of study participants with COVID-19 and a prolonged prothrombin time and elevated INR in 53.3% of study subjects with severe and 51 % of critically COVID patients. Thrombocytopenia was detected in 22.1% of COVID-19 patients. 50.5% and 51.3% of COVID-19 patients older than 55 years had thrombocytopenia and prolonged APTT respectively.

Conclusion:

In this study, prolonged prothrombin time and elevated INR were detected in more than 50% of severe and critical COVID-19 patients. Thrombocytopenia and prolonged APTT were dominant in COVID-19 patients older than 55 years. Thus, we recommend emphasis to be given for monitoring of platelet count, PT, APTT and INR in hospitalized and admitted COVID-19 patients.

Key words: activated partial thromboplastin time, COVID-19, Prothrombin time, international normalized ratio, Platelet, Addis Ababa, Ethiopia.

Introduction

Coronavirus disease 2019 (COVID-19) is caused by a novel beta corona virus called severe acute respiratory syndrome coronavirus 2 (SARS-CoV-2)(1). COVID-19 has become a pandemic that has affected the global population. As of November 8, 2020, there have been more than 49 million confirmed cases of COVID-19 and more than 1.2 million deaths, reported to World Health organization (WHO). Similarly, there have been 99,204 confirmed cases of COVID-19 with 1,518 deaths in Ethiopia(2).

The severity of COVID-19 infection ranges considerably from asymptomatic to life threatening, with lung injury being the main clinical manifestation. Most of the patients have a favorable prognosis, but some rapidly progress to severe respiratory distress syndrome, coagulation dysfunction and multiple organ failures (3, 4). Although the pathophysiology and the underlining mechanisms of clinical manifestations remain unclear, thrombo inflammation and cytokine storm are clearly established components in Severe Acute Respiratory Distress Syndrome (SARS) of COVID-19(5-8).

Coagulopathy and abnormal coagulation parameters were indicated among the most significant biomarkers of poor prognosis in COVID-19 patients (9-11). A retrospective cohort study conducted in Spain Madrid demonstrated that COVID-19 non-survivors had significantly ~~lower prothrombin time,~~ abnormal coagulation parameters such as prolonged PT, APTT, higher D-dimer and higher fibrinogen levels compared to survivors indicating coagulation parameters could be an efficient measure for predicting the prognosis of patients with SARS COV-2(7) ~~and used as~~ guiding clinical management. Similarly, Long et al has reported that occurrence of coagulation dysfunction is more likely in severe and critically ill patients. The study also showed that D-dimer and prothrombin time could be considered as main indicators in predicting the

mortality of COVID-19 patients (3). Many studies have also demonstrated the increased occurrence of intravascular disseminated coagulopathy (DIC) in patients with COVID-19 (12, 13). The result of blood coagulation profiles in COVID-19 patients can ~~also~~ guide management decisions and improve outcomes (12, 14).

Moreover, routine coagulation parameter tests results could potentially be utilized in symptomatic patients in resource limited settings with inadequate access to COVID-19 RT-PCR, as in Ethiopia, to raise suspension of this infection. However, data on coagulation profiles among Ethiopian COVID-19 patients is not readily available. Thus, the aim of this study was to determine the coagulation profile of COVID-19 patients admitted at Millennium COVID-19 treatment center, Addis Ababa, Ethiopia.

Methods

Ethical consideration: Ethical clearance was obtained and approved by Addis Ababa University College of Health Sciences, department of Medical Laboratory Sciences research ethics review committee (DRERC/538/20/MLS) and it was in accordance with the principles of the Helsinki II declaration. Laboratory test results were communicated to the responsible clinicians working at COVID-19 care and treatment center. Written informed consent was obtained from the study participants. All the personal identifying information obtained from the study participants were kept confidential.

Study population

In this study, we have included 455 consecutive patients with confirmed SARS-CoV-2 infection admitted to Millennium COVID-19 treatment center, Addis Ababa, Ethiopia from July 1-October 23, 2020. The treatment center is the first referral center for COVID-19 patients in

Ethiopia, since May 02, 2020. None of the study participants were receiving anticoagulant medications before blood drawing. Diagnosis of SARS-CoV-2 infection was made with real time RT-PCR.

Sample collection and coagulation profile analysis

2.6. Laboratory Analysis

Venous bloods were collected by professional nurses working in the COVID-19 care and treatment center: 5 mL in EDTA and 3 mL in 3.2% sodium citrated anti-coagulated tube for analysis of coagulation parameters. The samples for coagulation profile tests were collected at hospital admission. The prothrombin time (PT), activated partial prothrombin time (APTT), and international normalized ratio (INR) were analyzed using HUMACLOT DUE PLUS® coagulation analyzer (Wiesbaden ®, Germany). Platelet count was performed using UniCel® DxH 800 Coulter®Cellular Analysis System (Beckman Coulter ®, Inc. 4300 N. Harbor Blvd. Fullerton, CA 92835). The coagulation parameters were compared with the manufacturer cut off normal range of PT = 11.7- 15 seconds, APTT = 23.8- 37.9 seconds, INR = 1.0- 1.2 and PLT= 159-386/µ.l. The coagulation parameters above the cut off value were considered as a prolonged and thrombocytopenia in the case of lower than cut off value for platelets. All laboratory tests and interpretation were done following the manufacturers' recommendation and standard operating procedures set out by the center.

Statistical Analysis

Statistical Package for the Social Sciences (SPSS) software version 25.0 (SPSS® Inc., Chicago, IL, USA) was used for statistical analysis. Chi-square test was used to determine association

among categorical variables. The quantitative data were expressed as Mean ± SD. P value < 0.05 was considered as statistically significant.

Results

Socio-demographic and Clinical characteristics of Study participants

In this study, 455 patients diagnosed with COVID-19 were included. Among the study participants, 289 (63.5%) were males. The study participants were between the age of 4 and 90 years with a mean of 49.9 ±18.3 years. From the total 455 study subjects, there were 297 mild cases, 90 severe cases, and 68 critical cases based on disease severity of COVID-19(Table 1).

Table 1: Socio-demographic characteristics of study participants

Variables		Frequency	Percent
Gender	Male	289	63.5%
	Female	166	36.5%
Age group	0-18 years	15	3.2%
	18-35 years	101	22.1%
	36-55 years	158	34.7%
	>55 years	181	39.7%
Disease severity	Moderate	297	65.2%
	Severe	90	19.8%
	Critical	68	15%

The median time from the disease onset to admission was 4 days (2-8 days). Severe and critical groups showed differences in sex ratio and age distribution. In severe (36.6%) and critical groups (48.5%), were elderly males of the age of >55 years old. (Table 2).

Table 2: Socio-demographic characteristics and disease severity of COVID-19 patients

Variables		Disease Severity			P-value
		Moderate, n (%)	Severe, n (%)	Critical, n (%)	
Age(year)	0-18, n= 15	10(66.7)	4(26.7)	1(6.7)	0.283
	18-35, n=101	65(64.35)	22(21.78)	14(13.8)	
	36-55, n=158	107(67.7)	31(19.6)	20(12.65)	
	>55, n=181	115(63.5)	33(18.2)	33(8.2)	
Sex,	Male, n=289	187(64.7)	56(19.3)	46(15.9)	0.045
	Female, n=166	110(66.2)	34(20.4)	22(13.2)	

Magnitude of coagulation abnormalities

In this study, 209 COVID-19 patients (46%) showed prolonged PT and elevated INR values. Among those study participants with prolonged PT, 51.3% were above 55 years of age. Prolonged PT values were demonstrated more frequently among males (49.8%) than females (41%) and this difference was statistically significant (P = 0.045). Similarly, 51.4% and 53.3% of ICU (critical) and severe patients had prolonged PT values. Notably, prolonged APTT values were found among 43.1% of individuals, and from these 47%, 45% and 41% were among ICU (critical), severe and moderate patients, respectively. 57.2% of female patients had prolonged APTT; and 51.3% of patients aged older than 55 years had a prolonged APTT.

Thrombocytopenia was detected in 22.1% (101 out of 455) study subjects. 50.5% (50 out of 99) patients aged older than 55 years had thrombocytopenia and the occurrence was higher among male (23.8%) than female (18%) ICU patients (Table 3).

Table 3: Result of coagulation parameters in patients with severe COVID-19 according to different variables

Coagulation parameters		***Variables***								
		Age				***Sex***		***Disease severity***		
		0-18 n(%)	***19-35 n(%)***	***36-55 n(%)***	***>55 n(%)***	***Male n(%)***	***Female n(%)***	***Moderate n(%)***	***Severe n(%)***	***Critical n(%)***
PT	High n=213	9(4.2)	50(23.47)	61(28.6	93(43.6)	144(67.6)	69(32.4)	130(61)	48(22.5)	35(16.4)
	Normal n=220	6(2.7)	45(20.45)	89(40.4)	80(36.3)	131(59.5)	89(40.4)	149(67.7)	40(18.1)	31(14.1)
	Low=22	0	6(27.2)	8(36.3)	8(36.3)	14(63.6)	8(36.3)	18(81)	2(9)	2(9)
APTT	High=196	6(3)	46(23.4)	68(34.7)	76(38.77)	101(51.5)	95(48.5)	115(58.67)	41(21)	42(21.4)
	Normal n=193	6(3.1)	38(19.7)	70(36.2)	79(41)	136(70.4)	57(29.5)	137(71)	36(18.6)	21(10.8)
	Low n=66	3(4.5)	17(25.7)	20(30.3)	26(39.3)	52(78.7)	14(21)	45(68)	13(19.7)	5(7.5)
PLT	High n=65	4(6.1)	11(17)	24(37)	26(40)	43(66)	22(33.8)	39(60)	8(12.3)	8(12.3)
	Normal n=289	8(3)	70(24.2)	105(36.3)	105(36.3)	175(60.8)	114(39)	214(74)	44(15)	31(10.6)
	Low n= 101	3(2.9)	20(20)	28(27.7)	50(49.5)	69(69.70	30(30.3)	33(32)	38(37.6)	30(29.7)
INR	High n=210	9(4.2)	50(24.7)	60(28.5)	91(43.3)	141(67)	69(32.8)	127(60.4)	50(23.8)	33(15.7)
	Normal =224	5(2.2)	44(19.6)	93(41.5)	82(36.6)	115(51)	75(33.4)	113(50.4)	45(20)	32(14.2)
	Low n=21	1(4.7)	7(33.3)	5(23.8)	8(38)	14(66.6)	7(33.3)	15(71)	3(14.5)	3(14.5)

PLT=platelet; PT= prothrombin time; APTT=activated partial thromboplastin time; INR=international normalized ratio.

Discussion

The COVID-19 pandemic had a major impact on health care globally. COVID-19 has already caused >1.2 million deaths worldwide and more than 1400 in Ethiopia as of October 30,2020 according to WHO report(15). Coagulation abnormalities are frequent in COVID-19 patients and are associated with poor prognosis and reduced survival(7). The dysregulation of coagulation associated with hypercoagulability manifests as venous and arterial thrombosis and multiorgan dysfunction (16) which are poor prognostic markers (13, 14, 17-19). Previous studies indicated that the coagulopathy in patients hospitalized with COVID-19 is characterized by increases in coagulation parameters such as PT, APTT and INR (20, 21).

Patients with serious infection are more likely to have COVID-19 associated coagulopathy than patients with a mild infection (21, 22). In this recent study, prolonged PT, APTT and INR were more frequent among severe and critical COVID-19 patients. Similarly, studies also reported that thrombotic complications are common among COVID-19 patients admitted to intensive care unit (ICU) (22-24).

Treatment of the underlying condition is suggested to be paramount in coagulopathies. It is shown that bleeding is not common clinical manifestation in COVID-19 infections despite abnormal coagulation parameters (23,24) and supportive care including blood product transfusion should be individualized in COVID -19 patients(25, 26). Laboratory findings alone should not be taken as basis for instituting blood transfusion therapy, rather it should be reserved for those who are bleeding, requires an invasive procedure, or who are otherwise at high risk for bleeding complications (26, 27).

Published studies indicate that COVID-19 is associated with a hyper-coagulable state. Venous thromboembolism (VTE) and arterial thrombosis ranging from 15% to 30% were found in critically ill patients with COVID-19 and about 7% in those admitted to medical wards (28-30). Abnormal thrombosis of different medical devices, deep vein thrombosis and multiple thrombi in the vessels of the lungs, kidneys and other organs at autopsy of patients who died of Covid-19 have been reported serving as the impetus behind guidelines (9, 29)which support the use of therapeutic doses of heparin or low-molecular-weight heparin instead of prophylactic doses in critically ill COVID-19 patients (12, 26, 31).In the current study, thrombocytopenia was observed more frequently among males (23.8%) than females (19.8%) and older people (27.6%). Severe (42.68%) and critical (42%) patients also more frequently had thrombocytopenia and this was in line with studies conducted in different countries (20, 22, 32, 33). Thrombocytopenia, defined as platelet count less than 100×10^9 cells/L were independently associated with COVID-19 severity(34). Studies suggest that routine coagulation test results are markers of disease severity and assist in management decision. In critically ill patients, thrombocytopenia correlates with multi-organ failure and death, and a decline in platelet count, even in the absence of overt thrombocytopenia, portends a worse outcome (9, 12, 13). In patients who are not bleeding, there is no evidence that correction of laboratory parameters with blood products improves outcomes. Replacement might worsen disseminated thrombosis and further deplete scarce blood products (28, 35).

Many studies reported that coagulopathy associated with COVID-19 is characterized by thrombocytopenia, prolongation of the prothrombin time, high levels of D-dimer, and elevated levels of fibrinogen, factor VIII, and von Willebrand factor (3, 11, 16). Published studies indicate that COVID-19-associated coagulopathy is characterized by a decreased platelet count (9, 36-

38)and a cytokine storm with an extreme hyper-coagulable state. Even though the reason for this life-threatening condition is not known, this might be due to an uncontrolled hyper-inflammatory response without previous immunity (39, 40).

Conclusion: In this study, prolonged prothrombin time and high INR were found among severe and critical COVID-19 patients. Thrombocytopenia and prolonged clotting time assay were dominant in COVID-19 patients older than 55 years. Thus, we recommend emphasis to be given for monitoring of platelet count, PT, APTT and INR in hospitalized COVID-19 patients management.

Consent for publication: Not applicable

Availability of data and material

All the available data were included in the manuscript.

Funding: None

Conflict of interest: The authors declare that they have no conflict of interest.

References

1. Sohrabi C, Alsafi Z, O'Neill N, Khan M, Kerwan A, Al-Jabir A, et al. World Health Organization declares global emergency: A review of the 2019 novel coronavirus (COVID-19). Int J Surg. 2020;76:71-6.
2. World Health Organization. Who coronavirus disease (covid-19) dashboard 2020,November 8 [Available from: https://covid19.who.int/info.
3. Long H, Nie L, Xiang X, Li H, Zhang X, Fu X, et al. D-Dimer and Prothrombin Time Are the Significant Indicators of Severe COVID-19 and Poor Prognosis. BioMed research international. 2020;2020:6159720.
4. Guan WJ, Ni ZY, Hu Y, Liang WH, Ou CQ, He JX, et al. Clinical Characteristics of Coronavirus Disease 2019 in China. The New England journal of medicine. 2020;382(18):1708-20.
5. Aggarwal M, Dass J, Mahapatra M. Hemostatic Abnormalities in COVID-19: An Update. Indian J Hematol Blood Transfus. 2020:1-11.

6. Coccheri S. COVID-19: The crucial role of blood coagulation and fibrinolysis. Intern Emerg Med. 2020;15(8):1369-73.
7. Quintana-Díaz M, Andrés-Esteban EM, Ramírez-Cervantes KL, Olivan-Blázquez B, Juárez-Vela R, Gea-Caballero V. Coagulation Parameters: An Efficient Measure for Predicting the Prognosis and Clinical Management of Patients with COVID-19. Journal of Clinical Medicine. 2020;9(11).
8. Parasher A. COVID-19: Current understanding of its pathophysiology, clinical presentation and treatment. Postgrad Med J. 2020.
9. Al-Samkari H, Karp Leaf RS, Dzik WH, Carlson JCT, Fogerty AE, Waheed A, et al. COVID-19 and coagulation: bleeding and thrombotic manifestations of SARS-CoV-2 infection. Blood. 2020;136(4):489-500.
10. Annunziata A, Imitazione P, Polistina GE, Lanza M, Coppola A, Fiorentino G. Pulmonary Embolism in Covid-19: Coagulation Parameters, Close Monitoring to Prevent? Turk Thorac J. 2020;21(4):287-8.
11. Savioli F, Rocha LL. Coagulation profile in severe COVID-19 patients: what do we know so far? Revista Brasileira de Terapia Intensiva. 2020;32(2).
12. Buioni D, Nardi P, Ruvolo G. Thrombocytopenia and coagulation disorders due to COVID 19 infection with concomitant cardiovascular diseases requiring anti-platelet and anticoagulant therapy, which strategy? Clin Chim Acta. 2020;508:109.
13. Levi M. COVID-19 coagulopathy vs disseminated intravascular coagulation. Blood Adv. 2020;4(12):2850.
14. Quintana-Diaz M, Andres-Esteban EM, Ramirez-Cervantes KL, Olivan-Blazquez B, Juarez-Vela R, Gea-Caballero V. Coagulation Parameters: An Efficient Measure for Predicting the Prognosis and Clinical Management of Patients with COVID-19. J Clin Med. 2020;9(11).
15. WHO. COVID19. 2020. Contract No.:

.
16. Chan NC, Weitz JI. COVID-19 coagulopathy, thrombosis, and bleeding. Blood. 2020;41:100648.
17. Rauch A, Labreuche J, Lassalle F, Goutay J, Caplan M, Charbonnier L, et al. Coagulation biomarkers are independent predictors of increased oxygen requirements in COVID-19. J Thromb Haemost. 2020.
18. Zhang Y, He L, Chen H, Lu S, Xiong Y, Liu J, et al. Manifestations of blood coagulation and its relation to clinical outcomes in severe COVID-19 patients: Retrospective analysis. Int J Lab Hematol. 2020.
19. Zhang A, Leng Y, Zhang Y, Wu K, Ji Y, Lei S, et al. Meta-analysis of coagulation parameters associated with disease severity and poor prognosis of COVID-19. International journal of infectious diseases : IJID : official publication of the International Society for Infectious Diseases. 2020;100:441-8.
20. Luo HC, You CY, Lu SW, Fu YQ. Characteristics of coagulation alteration in patients with COVID-19. Ann Hematol. 2020.
21. Levi M, Thachil J, Iba T, Levy JH. Coagulation abnormalities and thrombosis in patients with COVID-19. Lancet Haematol. 2020;7(6):e438-e40.
22. Zou Y, Guo H, Zhang Y, Zhang Z, Liu Y, Wang J, et al. Analysis of coagulation parameters in patients with COVID-19 in Shanghai, China. Biosci Trends. 2020;14(4):285-9.

23. Sayad B, Rahimi Z. Blood coagulation parameters in patients with severe COVID-19 from Kermanshah Province, Islamic Republic of Iran. East Mediterr Health J. 2020;26(9):999-1004.
24. Adam EH, Zacharowski K, Miesbach W. A comprehensive assessment of the coagulation profile in critically ill COVID-19 patients. Thromb Res. 2020;194:42-4.
25. Paar V, Wernly B, Zhou Z, Motloch LJ, Hoppe UC, Egle A, et al. Anti-coagulation for COVID-19 treatment: both anti-thrombotic and anti-inflammatory? J Thromb Thrombolysis. 2020.
26. Belen-Apak FB, Sarialioglu F. Pulmonary intravascular coagulation in COVID-19: possible pathogenesis and recommendations on anticoagulant/thrombolytic therapy. J Thromb Thrombolysis. 2020;50(2):278-80.
27. Lee AY, Connors JM, Kreuziger LB, Murphy M, Gernsheimer T, Lin Y, et al. COVID-19 and Coagulopathy 2020, December 1 [Available from: https://www.hematology.org/COVID-19/COVID-19-and-coagulopathy.
28. Harenberg J, Favaloro E. COVID-19: progression of disease and intravascular coagulation - present status and future perspectives. Clin Chem Lab Med. 2020;58(7):1029-36.
29. Pizzi R, Gini G, Caiano L, Castelli B, Dotan N, Magni F, et al. Coagulation parameters and venous thromboembolism in patients with and without COVID-19 admitted to the Emergency Department for acute respiratory insufficiency. Thromb Res. 2020;196:209-12.
30. Voicu S, Delrue M, Chousterman BG, Stepanian A, Bonnin P, Malissin I, et al. Imbalance between procoagulant factors and natural coagulation inhibitors contributes to hypercoagulability in the critically ill COVID-19 patient: clinical implications. Eur Rev Med Pharmacol Sci. 2020;24(17):9161-8.
31. Mei H, Hu Y. [Characteristics, causes, diagnosis and treatment of coagulation dysfunction in patients with COVID-19]. Zhonghua Xue Ye Xue Za Zhi. 2020;41(3):185-91.
32. Bao C, Tao X, Cui W, Yi B, Pan T, Young KH, et al. SARS-CoV-2 induced thrombocytopenia as an important biomarker significantly correlated with abnormal coagulation function, increased intravascular blood clot risk and mortality in COVID-19 patients. Exp Hematol Oncol. 2020;9:16.
33. Xiong M, Liang X, Wei YD. Changes in blood coagulation in patients with severe coronavirus disease 2019 (COVID-19): a meta-analysis. Br J Haematol. 2020;189(6):1050-2.
34. Kander T. Coagulation disorder in COVID-19. The Lancet Haematology. 2020;7(9):e630-e2.
35. Song JC, Wang G, Zhang W, Zhang Y, Li WQ, Zhou Z, et al. Chinese expert consensus on diagnosis and treatment of coagulation dysfunction in COVID-19. Mil Med Res. 2020;7(1):19.
36. Chen X, Wang Q, Xu M, Li C. A Retrospective Analysis of the Coagulation Dysfunction in COVID-19 Patients. Clin Appl Thromb Hemost. 2020;26:1076029620964868.
37. Pavoni V, Gianesello L, Pazzi M, Stera C, Meconi T, Frigieri FC. Evaluation of coagulation function by rotation thromboelastometry in critically ill patients with severe COVID-19 pneumonia. J Thromb Thrombolysis. 2020;50(2):281-6.
38. Savioli F, Rocha LL. Coagulation profile in severe COVID-19 patients: what do we know so far? Rev Bras Ter Intensiva. 2020;32(2):197-9.
39. Jose RJ, Manuel A. COVID-19 cytokine storm: the interplay between inflammation and coagulation. Lancet Respir Med. 2020;8(6):e46-e7.

40. Kasinathan G, Sathar J. Haematological manifestations, mechanisms of thrombosis and anti-coagulation in COVID-19 disease: A review. Ann Med Surg (Lond). 2020;56:173-7.

Blood coagulation parameter abnormalities in hospitalized patients with confirmed COVID-19 in Ethiopia

Shambel Araya[1&2*], Mintesnot Aragaw Mamo [1&2], Yakob Gebregziabher Tsegay[3&4], Asegdew Atlaw[2], Aschalew Aytenew[2], Abebe Hordofa[2], Abebe Edao Negeso[1], MogesWordofa[1], Tirhas Niguse[1], Mahlet Cheru[1], Zemenu Tamir[1]

[1]Department of Medical Laboratory Sciences, College of Health Sciences, Addis Ababa University, Addis Ababa, Ethiopia

[2]Department of Medical Laboratory, Millennium COVID-19 Treatment and Care Centre, St. Paual Millennium Medical College, Addis Ababa, Ethiopia;

[3]Department of Medical Biotechnology, Institute of Biotechnology, University of Gondar, Gondar, Ethiopia.

[4]Department of Research and development center, College of Health Sciences, Defense University, Addis Ababa, Ethiopia.

Address:

1. Shambel Araya (corresponding author);shambelaraya8@gmail.com
2. Mintsnot Aragaw Mamo:mintsh2015@gmail.com
3. Yakob Gebreegziabher Tsegaye: yakobtsegay17@gmail.com
4. Asegdew Atlaw: asegdew21@gmail.com
5. AschalewAytenew:aschu9033@gmail.com
6. Abebe Hordofa: abuhordofa@gmail.com
7. Abebe EdaoNegeso:abenegesso@gmail.com
8. Moges Wordofa: heranmakmow@gmail.com
9. TirhasNiguse;peace.for.all.060610@gmail.com
10. MahletCheru:yuluyaya54@gmail.com
11. Zemenu Tamir: zemenut266@gmail.com

Abstract

Background: Coagulopathy and thromboembolic events are among the complications of Corona Virus disease 2019 (COVID-19). ~~Thus, a~~Abnormal coagulation ~~profiles~~ parameters in COVID-19 patients are ~~taken as~~ important prognostic factors of ~~COVID-19~~ disease severity. The aim of this study was to analyze coagulation profiles of hospitalized COVID-19 patients in Addis Ababa, Ethiopia. **Methods**: This prospective cross-sectional study was conducted among 455 Covid-19 patients admitted at Millennium COVID-19 care and treatment center, Addis Ababa, Ethiopia from July 1- October 23, 2020. Prothrombin Time (PT), Activated Partial Thromboplastin Time (APTT) and International normalized ratio (INR) were determined on HUMACLOT DUE PLUS® coagulation analyzer (Wiesbaden, Germany). In all statistical analysis of results ~~tests~~, p<0.05 was defined as statistically significant.

Result: A prolonged prothrombin time was found in 46.8% of study ~~subjects~~ ~~participants~~ ~~with~~participants with COVID-19 and a prolonged prothrombin time and elevated INR in~~with~~ 53.3% of study subjects with severe and 51 % of critically COVID patients. Thrombocytopenia was detected in 22.1% of COVID-19 patients. 50.5% and 51.3% of COVID-19 patients older than 55 years had thrombocytopenia and prolonged APTT respectively.

Conclusion:

In this study, prolonged prothrombin time and ~~high~~ elevated INR were detected in more than~~around~~ 50% of severe and critical COVID-19 patients. Thrombocytopenia and prolonged APTT were dominant in COVID-19 patients older than 55 years. Thus, we recommend emphasis to be given for monitoring of platelet count, PT, APTT and INR in hospitalized and admitted COVID-19 patients.

Key words: activated partial thromboplastin time~~, COVID~~, COVID-19, Prothrombin time, ~~activated partial thromboplastin time~~, international normalized ratio, Platelet, Addis Ababa, Ethiopia.

Introduction

Coronavirus disease 2019 (COVID-19) is caused by a novel beta corona virus called severe acute respiratory syndrome coronavirus 2 (SARS-CoV-2)(1). COVID-19 has become a pandemic that has ~~heavily~~ affected the global population. As of November 8, 2020, there have been more than 49 million ~~49,578,590~~ confirmed cases of COVID-19 and more than 1.2 million~~1,245,717~~ deaths, reported to World Health organization (WHO). Similarly, there have been 99,204 confirmed cases of COVID-19 with 1,518 deaths in Ethiopia(2).

The severity of COVID-19 infection ranges ~~varies~~ considerably from asymptomatic to life threatening, with lung injury being the main clinical manifestation. Most of the patients have a favorable prognosis, but some rapidly progress to severe ~~and critical cases with~~ respiratory distress syndrome, coagulation dysfunction and multiple organ failures (3, 4). Although the pathophysiology and the underlining mechanisms of clinical manifestations remain unclear, thrombo-inflammation and cytokine storm are clearly established components in Severe Acute Respiratory Distress Syndrome (SARS) of COVID-19(5-8).

Coagulopathy and abnormal coagulation ~~profiles~~ parameters were indicated among the most significant biomarkers of poor prognosis in COVID-19 patients (9-11). A retrospective cohort study conducted in Spain Madrid demonstrated that COVID-19 non-survivors had significantly lower prothrombin ~~activity~~time, abnormal coagulation parameters ~~like~~ such as prolonged PT, APTT, higher D- dimer and higher fibrinogen levels compared to survivors indicating coagulation parameters could be an efficient measure for predicting the prognosis of patients with SARS COV-2(7) and used as guiding clinical management. Similarly, Long et al has reported that occurrence of coagulation dysfunction is more likely in severe and critically ill patients. The study also showed that D-dimer and prothrombin time could be considered as main

indicators in predicting the mortality of COVID-19 patients (3). ~~Several~~ M~~m~~any studies have also demonstrated the increased occurrence of intravascular disseminated coagulopathy (DIC) in patients with COVID-19 (12, 13). The result of blood coagulation ~~parameters~~ profiles in COVID-19 patients can also guide management decisions and improve outcomes (12, 14).

Moreover, routine coagulation parameter tests results could potentially be utilized in symptomatic patients in resource limited settings with inadequate access to COVID-19 RT-PCR, as in Ethiopia, to raise suspension of this infection. However, data on coagulation profiles among Ethiopian COVID-19 patients is not readily available. Thus, the aim of this study was to determinethe coagulation profile of COVID-19 patients admitted at Millennium COVID-19 treatment center, Addis Ababa, Ethiopia.

Methods

Ethical consideration: Ethical clearance was obtained and approved by Addis Ababa University College of Health Sciences, department of Medical Laboratory Sciences research ethics review committee (DRERC/538/20/MLS) and it was in accordance with the principles of the Helsinki II declaration. Laboratory test results were communicated to the responsible clinicians working at COVID-19 care and ~~the~~ treatment center. Written informed consent was obtained from the study participants. All the personal identifying information obtained from the study participants were kept confidential.

Study population

In this study, we have included 455 consecutive patients with confirmed SARS-CoV-2 infection admitted to Millennium COVID-19 treatment center, Addis Ababa, Ethiopia from July 1-October 23, 2020. The treatment center is the first referral center for COVID-19 patients in

Ethiopia, since May 02, 2020. None of the study participants were ~~receving~~receiving anticoagulant medications before blood drawing. Diagnosis of SARS-CoV-2 infection was made with real time RT-PCR.

Sample collection and coagulation profile analysis

2.6. Laboratory Analysis

Venous bloods were collected by professional nurses working in the COVID-19 care and treatment center: 5 mL in EDTA and 3 mL in 3.2% sodium citrated anti-coagulated tube for analysis of coagulation parameters. The samples for coagulation profile tests were collected at hospital admission. The prothrombin time (PT), activated partial prothrombin time (APTT), and international normalized ratio (INR) were analyzed using HUMACLOT DUE PLUS® coagulation analyzer (Wiesbaden ®, Germany). Platelet count was performed using UniCel® DxH 800 Coulter®Cellular Analysis System (Beckman Coulter ®, Inc. 4300 N. Harbor Blvd. Fullerton, CA 92835). The coagulation parameters were compared with the manufacturer cut off normal range of PT = 11.7- 15 seconds, APTT = 23.8- 37.9 seconds, INR = 1.0- 1.2 and PLT= 159-386/µ.l. The coagulation parameters above the cut off ~~range~~ value were considered as a prolonged ~~time~~ and thrombocytopenia in the case of lower than cut off value for platelets. All laboratory tests and ~~its~~ interpretation were done following the manufacturers' recommendation and standard operating procedures set out by the center.

Statistical Analysis

Statistical Package for the Social Sciences (SPSS) software version 25.0 (SPSS® Inc., Chicago, IL, USA) was used for statistical analysis. Chi-square test was used to determine association

among categorical variables. The quantitative data were expressed as Mean ± SD. P value < 0.05 was considered as statistically significant.

Results

Socio-demographic and Clinical characteristics of Study participants

In this study, 455 patients diagnosed with COVID-19 were included. Among the study participants, 289 (63.5%) were males. The study participants were between the age of 4 and 90 years with a mean of 49.9 ±18.3 years. From the total 455 ~~case~~study ~~subjects ,~~subjects, there were 297 mild cases, 90 severe cases, and 68 critical cases based on disease severity of COVID-19(Table 1).

Table 1: Socio-demographic characteristics of study participants

Variables		Frequency	Percent
Gender	Male	289	63.5%
	Female	166	36.5%
Age group	0-18 years	15	3.2%
	18-35 years	101	22.1%
	36-55 years	158	34.7%
	>55 years	181	39.7%
Disease severity	Moderate	297	65.2%

	Severe	90	19.8%
	Critical	68	15%

The median time from the disease onset to admission was 4 days (2-8 days). Severe and critical groups showed differences in sex ratio and age distribution. In severe (36.6%) and critical groups (48.5%), were elderly males of the age of >55 years old. ~~y~~ (Table 2).

Table 2: Socio-demographic characteristics and disease severity of COVID-19 patients

Variables		Disease Severity			P-value
		Moderate, n (%)	Severe, n (%)	Critical, n (%)	
Age(year)	0-18, n= 15	10(66.7)	4(26.7)	1(6.7)	0.283
	18-35, n=101	65(64.35)	22(21.78)	14(13.8)	
	36-55, n=158	107(67.7)	31(19.6)	20(12.65)	
	>55, n=181	115(63.5)	33(18.2)	33(8.2)	
Sex,	Male, n=289	187(64.7)	56(19.3)	46(15.9)	0.045
~~N=455~~	Female, n=166	110(66.2)	34(20.4)	22(13.2)	

Magnitude of coagulation abnormalities

In this study, 209 COVID-19 patients (46%) showed prolonged PT and elevated~~higher~~ INR values. Among those ~~patients~~ study participants with prolonged PT, 51.3% were above 55 years of age. Prolonged PT values were demonstrated more frequently among males (49.8%) than females (41%) and this difference was statistically ~~different~~significant (P = 0.045). Similarly, 51.4% and 53.3% of ICU (critical) and severe patients had prolonged PT values. Notably, prolonged APTT values were found among 43.1% of individuals, and from these 47%, 45% and

41% were among ICU (critical), severe and moderate patients, respectively. 57.2% of female patients had prolonged APTT; and 51.3% of patients aged older than 55 years had a prolonged APTT.

Thrombocytopenia was detected in 22.1% (101 out of 455) study subjects. 50.5% (50 out of 99) patients aged older than 55 years had thrombocytopenia and the occurrence was higher among male (23.8%) than female (18%) ICU patients (Table 3).

Coagulation Parameters		***Variables***								
		Age				***Sex***		***Disease Severity***		
		0-18 n(%)	***19-35 n(%)***	***36-55 n(%)***	***>55 n(%)***	***Male n(%)***	***Female n(%)***	***Moderate n(%)***	***Severe n(%)***	***Critical n(%)***
PT	High n=213	9(4.2)	50(23.47)	61(28.6	93(43.6)	144(67.6)	69(32.4)	130(61)	48(22.5)	35(16.4)
	Normal n=220	6(2.7)	45(20.45)	89(40.4)	80(36.3)	131(59.5)	89(40.4)	149(67.7)	40(18.1)	31(14.1)
	Low=22	0	6(27.2)	8(36.3)	8(36.3)	14(63.6)	8(36.3)	18(81)	2(9)	2(9)
APTT	High=196	6(3)	46(23.4)	68(34.7)	76(38.77)	101(51.5)	95(48.5)	115(58.67)	41(21)	42(21.4)
	Normal n=193	6(3.1)	38(19.7)	70(36.2)	79(41)	136(70.4)	57(29.5)	137(71)	36(18.6)	21(10.8)
	Low n=66	3(4.5)	17(25.7)	20(30.3)	26(39.3)	52(78.7)	14(21)	45(68)	13(19.7)	5(7.5)
PLT	High n=65	4(6.1)	11(17)	24(37)	26(40)	43(66)	22(33.8)	39(60)	8(12.3)	8(12.3)

	Normal n=289	8(3)	70(24.2)	105(36.3)	105(36.3)	175(60.8)	114(39)	214(74)	44(15)	31(10.6)
	Low n= 101	3(2.9)	20(20)	28(27.7)	50(49.5)	69(69.70	30(30.3)	33(32)	38(37.6)	30(29.7)
INR	High n=210	9(4.2)	50(24.7)	60(28.5)	91(43.3)	141(67)	69(32.8)	127(60.4)	50(23.8)	33(15.7)
	Normal =224	5(2.2)	44(19.6)	93(41.5)	82(36.6)	115(51)	75(33.4)	113(50.4)	45(20)	32(14.2)
	Low n=21	1(4.7)	7(33.3)	5(23.8)	8(38)	14(66.6)	7(33.3)	15(71)	3(14.5)	3(14.5)

Table 3: Result of coagulation parameters in patients with severe COVID-19 according to different variables

PLT=platelet; PT= prothrombin time; APTT=activated partial thromboplastin time; INR=international normalized ratio.

Discussion

The COVID-19 pandemic had a major impact on health care globally. COVID-19 has already caused >1.2 million deaths worldwide and more than 1400 in Ethiopia as of October 30,2020 according to WHO report(15). Coagulation abnormalities are frequent in COVID-19 patients and are associated with poor prognosis and reduced survival(7). The dysregulation of coagulation ~~and~~ associated with hypercoagulability ~~in patients with COVID~~ manifests as venous and arterial thrombosis and multiorgan dysfunction(16)~~;~~ which are poor prognostic markers ~~resulting in increased mortality and hospitalization and ICU admission~~ (13, 14, 17-19). Previous studies indicated that the coagulopathy in patients hospitalized with COVID-19 is characterized by increases in coagulation parameters such as PT, APTT and INR (20, 21).

Patients with serious infection are more likely to have COVID-19 associated coagulopathy than patients with a mild infection (21,22). ~~In our~~In thise recent study, prolonged PT, APTT and INR were~~was~~ more frequent among severe and critical COVID-19 patients. Similarly, studies also reported that thrombotic complications are common among COVID-19 patients admitted to intensive care unit (ICU) ~~(9.5%-47%)~~(22-24).

Treatment of the underlying condition is suggested to be paramount in coagulopathies. It is shown that bleeding is not common clinical manifestation in COVID-19 infections despite abnormal coagulation parameters (23,24)~~.~~ and supportive care including blood product transfusion should be individualized in COVID -19 patients(25, 26). Laboratory findings alone should not be taken as basis for instituting blood transfusion therapy, rather it should be reserved for those who are bleeding, requires an invasive procedure, or who are otherwise at high risk for bleeding complications (26, 27).

Published studies indicate that COVID-19 is associated with a hyper-coagulable state. Venous thromboembolism (VTE) and arterial thrombosis ranging from 15% to 30% were found in critically ill patients with COVID-19 and about 7% in those admitted to medical wards (28-30). Abnormal thrombosis of different medical devices, deep vein thrombosis and multiple thrombi in the vessels of the lungs, kidneys and other organs at autopsy of patients who died of Covid-19 have been reported serving as the impetus behind guidelines (9, 29)which support the use of therapeutic doses of heparin or low-molecular-weight heparin instead of prophylactic doses in critically ill COVID-19 patients (12, 26, 31).In the current study, thrombocytopenia was observed more frequently among males (23.8%) than females (19.8%) and older people (27.6%). Severe (42.68%) and critical (42%) patients also more frequently had thrombocytopenia and this was in line with studies conducted in different countries (20, 22, 32, 33). Thrombocytopenia,

defined as platelet count less than 100×10^9 cells/L were independently associated with COVID-19 severity(34). Studies suggest that routine coagulation test results are markers of disease severity and assist in management decision.In critically ill patients, thrombocytopenia correlates with multi-organ failure and death, and a decline in platelet count, even in the absence of overt thrombocytopenia, portends a worse outcome (9, 12, 13). In patients who are not bleeding, there is no evidence that correction of laboratory parameters with blood products improves outcomes. Replacement might worsen disseminated thrombosis and further deplete scarce blood products (28, 35).

Many studies reported that coagulopathy associated with COVID-19 is characterized by thrombocytopenia, prolongation of the prothrombin time, high levels of D-dimer, and elevated levels of fibrinogen, factor VIII, and von Willebrand factor (3, 11, 16). The degree of coagulation abnormalities in critically ill non COVID patients correlates with disease severity and predict the risk of thrombosis, the need for ventilator support, and mortality. Published studies indicatesupport that COVID-19-associated coagulopathy is characterized by a decreased platelet count (9, 36-38). Patients with critical COVID-19 infection and a cytokine storm withhave an extreme hyper-coagulable state. Even though the reason for this life-threatening condition is not known, this might be due to an uncontrolled hyper-inflammatory response without previous immunity (39, 40).

Conclusion: In this study, prolonged prothrombin time and high INR were found among severe and critical COVID-19 patients. Thrombocytopenia and prolonged clotting time assay APTT were dominant in COVID-19 patients older than 55 years. Thus, we recommend emphasis to be

given for monitoring of platelet count, PT, APTT and INR in hospitalized COVID-19 patients management.

Consent for publication: Not applicable

Availability of data and material

All the available data were included in the manuscript.

Funding: None

Conflictof interest: The authors declare that they have no conflict of interest.

References

1. Sohrabi C, Alsafi Z, O'Neill N, Khan M, Kerwan A, Al-Jabir A, et al. World Health Organization declares global emergency: A review of the 2019 novel coronavirus (COVID-19). Int J Surg. 2020;76:71-6.
2. World Health Organization. Who coronavirus disease (covid-19) dashboard 2020,November 8 [Available from: https://covid19.who.int/info.
3. Long H, Nie L, Xiang X, Li H, Zhang X, Fu X, et al. D-Dimer and Prothrombin Time Are the Significant Indicators of Severe COVID-19 and Poor Prognosis. BioMed research international. 2020;2020:6159720.
4. Guan WJ, Ni ZY, Hu Y, Liang WH, Ou CQ, He JX, et al. Clinical Characteristics of Coronavirus Disease 2019 in China. The New England journal of medicine. 2020;382(18):1708-20.
5. Aggarwal M, Dass J, Mahapatra M. Hemostatic Abnormalities in COVID-19: An Update. Indian J Hematol Blood Transfus. 2020:1-11.
6. Coccheri S. COVID-19: The crucial role of blood coagulation and fibrinolysis. Intern Emerg Med. 2020;15(8):1369-73.
7. Quintana-Díaz M, Andrés-Esteban EM, Ramírez-Cervantes KL, Olivan-Blázquez B, Juárez-Vela R, Gea-Caballero V. Coagulation Parameters: An Efficient Measure for Predicting the Prognosis and Clinical Management of Patients with COVID-19. Journal of Clinical Medicine. 2020;9(11).
8. Parasher A. COVID-19: Current understanding of its pathophysiology, clinical presentation and treatment. Postgrad Med J. 2020.
9. Al-Samkari H, Karp Leaf RS, Dzik WH, Carlson JCT, Fogerty AE, Waheed A, et al. COVID-19 and coagulation: bleeding and thrombotic manifestations of SARS-CoV-2 infection. Blood. 2020;136(4):489-500.
10. Annunziata A, Imitazione P, Polistina GE, Lanza M, Coppola A, Fiorentino G. Pulmonary Embolism in Covid-19: Coagulation Parameters, Close Monitoring to Prevent? Turk Thorac J. 2020;21(4):287-8.

11. Savioli F, Rocha LL. Coagulation profile in severe COVID-19 patients: what do we know so far? Revista Brasileira de Terapia Intensiva. 2020;32(2).
12. Buioni D, Nardi P, Ruvolo G. Thrombocytopenia and coagulation disorders due to COVID 19 infection with concomitant cardiovascular diseases requiring anti-platelet and anticoagulant therapy, which strategy? Clin Chim Acta. 2020;508:109.
13. Levi M. COVID-19 coagulopathy vs disseminated intravascular coagulation. Blood Adv. 2020;4(12):2850.
14. Quintana-Diaz M, Andres-Esteban EM, Ramirez-Cervantes KL, Olivan-Blazquez B, Juarez-Vela R, Gea-Caballero V. Coagulation Parameters: An Efficient Measure for Predicting the Prognosis and Clinical Management of Patients with COVID-19. J Clin Med. 2020;9(11).
15. WHO. COVID19. 2020. Contract No.:

.
16. Chan NC, Weitz JI. COVID-19 coagulopathy, thrombosis, and bleeding. Blood. 2020;41:100648.
17. Rauch A, Labreuche J, Lassalle F, Goutay J, Caplan M, Charbonnier L, et al. Coagulation biomarkers are independent predictors of increased oxygen requirements in COVID-19. J Thromb Haemost. 2020.
18. Zhang Y, He L, Chen H, Lu S, Xiong Y, Liu J, et al. Manifestations of blood coagulation and its relation to clinical outcomes in severe COVID-19 patients: Retrospective analysis. Int J Lab Hematol. 2020.
19. Zhang A, Leng Y, Zhang Y, Wu K, Ji Y, Lei S, et al. Meta-analysis of coagulation parameters associated with disease severity and poor prognosis of COVID-19. International journal of infectious diseases : IJID : official publication of the International Society for Infectious Diseases. 2020;100:441-8.
20. Luo HC, You CY, Lu SW, Fu YQ. Characteristics of coagulation alteration in patients with COVID-19. Ann Hematol. 2020.
21. Levi M, Thachil J, Iba T, Levy JH. Coagulation abnormalities and thrombosis in patients with COVID-19. Lancet Haematol. 2020;7(6):e438-e40.
22. Zou Y, Guo H, Zhang Y, Zhang Z, Liu Y, Wang J, et al. Analysis of coagulation parameters in patients with COVID-19 in Shanghai, China. Biosci Trends. 2020;14(4):285-9.
23. Sayad B, Rahimi Z. Blood coagulation parameters in patients with severe COVID-19 from Kermanshah Province, Islamic Republic of Iran. East Mediterr Health J. 2020;26(9):999-1004.
24. Adam EH, Zacharowski K, Miesbach W. A comprehensive assessment of the coagulation profile in critically ill COVID-19 patients. Thromb Res. 2020;194:42-4.
25. Paar V, Wernly B, Zhou Z, Motloch LJ, Hoppe UC, Egle A, et al. Anti-coagulation for COVID-19 treatment: both anti-thrombotic and anti-inflammatory? J Thromb Thrombolysis. 2020.
26. Belen-Apak FB, Sarialioglu F. Pulmonary intravascular coagulation in COVID-19: possible pathogenesis and recommendations on anticoagulant/thrombolytic therapy. J Thromb Thrombolysis. 2020;50(2):278-80.
27. Lee AY, Connors JM, Kreuziger LB, Murphy M, Gernsheimer T, Lin Y, et al. COVID-19 and Coagulopathy 2020, December 1 [Available from: https://www.hematology.org/COVID-19/COVID-19-and-coagulopathy.
28. Harenberg J, Favaloro E. COVID-19: progression of disease and intravascular coagulation - present status and future perspectives. Clin Chem Lab Med. 2020;58(7):1029-36.

29. Pizzi R, Gini G, Caiano L, Castelli B, Dotan N, Magni F, et al. Coagulation parameters and venous thromboembolism in patients with and without COVID-19 admitted to the Emergency Department for acute respiratory insufficiency. Thromb Res. 2020;196:209-12.
30. Voicu S, Delrue M, Chousterman BG, Stepanian A, Bonnin P, Malissin I, et al. Imbalance between procoagulant factors and natural coagulation inhibitors contributes to hypercoagulability in the critically ill COVID-19 patient: clinical implications. Eur Rev Med Pharmacol Sci. 2020;24(17):9161-8.
31. Mei H, Hu Y. [Characteristics, causes, diagnosis and treatment of coagulation dysfunction in patients with COVID-19]. Zhonghua Xue Ye Xue Za Zhi. 2020;41(3):185-91.
32. Bao C, Tao X, Cui W, Yi B, Pan T, Young KH, et al. SARS-CoV-2 induced thrombocytopenia as an important biomarker significantly correlated with abnormal coagulation function, increased intravascular blood clot risk and mortality in COVID-19 patients. Exp Hematol Oncol. 2020;9:16.
33. Xiong M, Liang X, Wei YD. Changes in blood coagulation in patients with severe coronavirus disease 2019 (COVID-19): a meta-analysis. Br J Haematol. 2020;189(6):1050-2.
34. Kander T. Coagulation disorder in COVID-19. The Lancet Haematology. 2020;7(9):e630-e2.
35. Song JC, Wang G, Zhang W, Zhang Y, Li WQ, Zhou Z, et al. Chinese expert consensus on diagnosis and treatment of coagulation dysfunction in COVID-19. Mil Med Res. 2020;7(1):19.
36. Chen X, Wang Q, Xu M, Li C. A Retrospective Analysis of the Coagulation Dysfunction in COVID-19 Patients. Clin Appl Thromb Hemost. 2020;26:1076029620964868.
37. Pavoni V, Gianesello L, Pazzi M, Stera C, Meconi T, Frigieri FC. Evaluation of coagulation function by rotation thromboelastometry in critically ill patients with severe COVID-19 pneumonia. J Thromb Thrombolysis. 2020;50(2):281-6.
38. Savioli F, Rocha LL. Coagulation profile in severe COVID-19 patients: what do we know so far? Rev Bras Ter Intensiva. 2020;32(2):197-9.
39. Jose RJ, Manuel A. COVID-19 cytokine storm: the interplay between inflammation and coagulation. Lancet Respir Med. 2020;8(6):e46-e7.
40. Kasinathan G, Sathar J. Haematological manifestations, mechanisms of thrombosis and anti-coagulation in COVID-19 disease: A review. Ann Med Surg (Lond). 2020;56:173-7.
